# Okanin Suppresses the Growth of Colorectal Cancer Cells by Targeting at Peroxiredoxin 5

**DOI:** 10.1002/advs.202417148

**Published:** 2025-08-20

**Authors:** Ji Zhong Zhao, Yuan Fei Li, Fu Kang Yuan, Meng Lu Zhao, Ya Wen Han, Jia Xin Wang, Qi Yang, Han Ying Ye, Yu Cheng Lu, Shao Chin Lee

**Affiliations:** ^1^ Lab of Cell Biology School of Life Sciences Jiangsu Normal University Xuzhou Jiangsu 221116 P. R. China; ^2^ Department of Oncology the First Hospital of Shanxi Medical University Taiyuan Shanxi 030001 P. R. China; ^3^ Department of Surgery Xuzhou Central Hospital Xuzhou Jiangsu 221116 P. R. China; ^4^ Biobank Linyi People's Hospital Linyi Shandong 276000 P. R. China

**Keywords:** apoptosis, ferroptosis, okanin, PRDX5, ubiquitination

## Abstract

Okanin is a natural product with few known biological activities. Its anti‐cancer effects and the underlying mechanisms are investigated. It is found that okanin inhibits cancer cell growth (25–50 µm) with minimal effects on non‐cancerous colorectal cells except at much higher doses (i.e., > 100 µm). In colorectal HCT116 cancer cells, okanin binds directly to peroxiredoxin 5 (PRDX5) at a site opposite the catalytic domain, which directly inhibits the enzymatic activity and triggers the production of reactive oxygen species, leading to independent apoptosis and ferroptosis. The binding also causes WSB1‐mediated ubiquitination degradation of PRDX5, resulting in reduced transcription and SIAH2‐mediated ubiquitination degradation of GPX4, which similarly causes apoptosis and ferroptosis. In xenograft mouse models, okanin decreases the PRDX5 level and inhibits the growth of HCT116 cells, both of which are compromised when cells stably overexpressing PRDX5 are used. Okanin does not change the body weight of the animals; in comparison, 5‐fluorouracil reduces the body weight, despite being less effective. In conclusion, the results suggest that okanin targets PRDX5, which capacitates it for anti‐cancer activity via apoptosis and ferroptosis independently. Okanin is a promising investigational drug. PRDX5 and GPX4 are candidate targets for cancer chemotherapy, at least for colorectal cancer.

## Graphical abstract

Okanin suppresses colorectal cancer growth by directly targeting PRDX5. This natural compound selectively binds peroxiredoxin 5 (PRDX5), inhibiting its activity and inducing WSB1‐mediated degradation. PRDX5 loss elevates ROS, suppresses GPX4 via SIAH2, and further triggers cell death (apoptosis and ferroptosis). The results unveil a novel PRDX5/GPX4/SIAH2/WSB1 signaling axis as a promising therapeutic vulnerability for cancer.

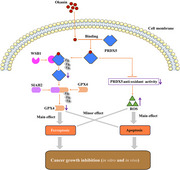

## 1 Introduction

Despite the advances in the past, cancer still remains the second leading cause of death worldwide after cardiovascular disease; according to the WHO, in 2020, there were more than 19.3 million new cases diagnosed and ≈10 million cancer‐related deaths. Surgery, chemotherapy, and radiotherapy are frontline treatments, with chemotherapy being a common option.^[^ ^1^ ^]^ However, the current chemotherapeutic protocols are often unable to deliver satisfactory outcomes, mostly due to dose‐limiting effects. Therefore, there is a need for the development of new chemotherapies. Natural products provide a broad resource for drug development. From 1981 to 2019, ≈60% of the drugs approved by the FDA were derived from natural products. More specifically, in 2016, 77% of the cancer chemotherapeutic agents were sourced from or based on natural compounds.^[^ ^2^ ^]^ Although targeted therapies have improved since the 1990s, the rapid emergence of drug resistance has limited their use. As a result, natural products have regained prominence in the development of anti‐cancer drugs.^[^ ^3^ ^]^ Compared to synthetic agents, natural products have a higher level of biological tolerance, as well as fewer side effects and less drug resistance,^[^ ^4^ ^]^ some of which are even capable of overcoming drug resistance.^[^ ^5^ ^]^

In a pilot screening study, we identified some compounds that could inhibit cancer cell viability in vitro, including okanin, which is a flavonoid monomer abundant in *Bidens bipinnata Linn*. and *Coreopsis tinctoria Nutt.’s*. These plants are used to treat a variety of diseases that include inflammation, hypertension, and diabetes in Traditional Chinese Medicine. Okanin itself has been demonstrated to inhibit blood coagulation and microglia activation,^[^ ^6^ ^]^ inflammation^[^ ^6^ ^]^ and sepsis.^[^ ^7^ ^]^

Programmed cell death, which determines the fate of cells and is implicated in the development of cancer,^[^ ^8^ ^]^ is a cell biology mechanism commonly underlying chemotherapy. Apoptosis is probably the best studied form of cell death with the intrinsic and extrinsic main pathways, in which caspase activation is a common feature. Ferroptosis is a comparatively newly identified non‐apoptotic form of cell death that is characterized by the accumulation of Fe^2+^ and lipid peroxides as well as decreased levels of cysteine glutathione and glutathione peroxidase 4 (GPX4).^[^ ^9^ ^]^ GPX4 is a member of the GPX family, which consists of 8 members, from GPX1 to GPX8. It is an essential selenoprotein responsible for reducing phospholipid hydroperoxide and is crucial in protecting cells from lipid peroxidation and ferroptosis.^[^ ^10^ ^]^ GPX4 is regulated at both the mRNA and protein levels; for example, NRF2 raises GPX4 mRNA^[^ ^11^ ^]^ and CST1 increases the stability of GPX4 protein.^[^ ^12^ ^]^ Notably, upregulation of GPX4 enhances the viability and drug‐resistance of cancer cells.^[^ ^13^ ^]^ It is believed that ferroptosis is also a cellular mechanism underlying chemotherapy, particularly for aggressive cancers that are unresponsive to traditional treatments.^[^ ^9^ ^]^ Biologically, cancer is a multi‐faceted disease comprised of genetic, epigenetic, metabolic, and signaling aberrations that severely disrupt the normal homeostasis of cell growth and death. Thus, clinically, multi‐targeted therapy such as combinational therapy is considered to be most effective, likely due to simultaneously targeting at different cell death pathways.^[^ ^14^ ^]^ Importantly, there are cases in which one compound can activate more than one signaling pathways to cause cell death; these “pro‐drugs” therefore have advantageous for the management of cancer and deserve chemotherapeutic development with priority.^[^ ^15^ ^]^

Peroxiredoxin 5 (PRDX5) is a member of a family that has six members (PRDX1‐PRDX6).^[^ ^16^ ^]^ It is an antioxidant enzyme that protects cells from oxidative stress by reducing hydrogen peroxide and alkyl hydroperoxides with reducing power equivalents to Trx system, with 10^5^ times more efficient at reducing H_2_O_2_ than free cysteine.^[^ ^17^ ^]^ In addition, this protein also plays a role in intracellular redox signaling. The N‐terminus of PRDX5 binds to other transcription factors, such as NRF2, which leads to the upregulation of NAPDH and attenuation of apoptosis via scavenging free radicals in non‐small‐cell lung cancer cells.^[^ ^18^ ^]^ There is evidence that PRDX5 is increased in castration‐resistant prostate cancer cells, in which it mediates resistance to treatment by androgen receptor inhibitors.^[^ ^19^ ^]^ The family member PRDX1 is a target of celastrol in the induction of colorectal cancer cell apoptosis.^[^ ^20^ ^]^

WD repeat and SOCS box containing 1 (WSB1) is a member of the elongin B/C‐cullin 2/5‐SOCS box containing ubiquitin ligase protein complex,^[^ ^21^ ^]^ which can promote tumor development of lung and breast cancer cells by inducing pVHL degradation^[^ ^22^ ^]^ as well as osteosarcoma cells by inducing Rho GDP dissociation inhibitor 2 degradation.^[^ ^23^ ^]^ Seven in absentia homolog 2 (SIAH2) is an RING E3 ubiquitin ligase, the role of which is controversial in cancer development.^[^ ^24^ ^]^ On one hand, its overexpression is associated with the tumorigenesis and progression of different carcinomas, including prostate,^[^ ^25^ ^]^ lung,^[^ ^26^ ^]^ and ovarian cancer.^[^ ^27^ ^]^ On the other hand, SIAH2 also ubiquitinates and degrades multiple oncoproteins, indicative of its potential role as a tumor suppressor, which includes targeting of tyrosine kinase 2 in lung cancer^[^ ^28^ ^]^; activated CDC42‐associated kinase 1 in breast cancer^[^ ^29^ ^]^; and promyelocytic leukemia–retinoic acid receptor α in leukemia.^[^ ^30^ ^]^

In the present study, we investigated the effects of okanin on the growth and death of HCT116 colorectal cancer cells in vitro and in vivo, as well as the underlying cellular and molecular mechanisms.

## 2 Results

### 2.1 Okanin Inhibits the Growth Colorectal Cancer Cells In Vitro and In Vivo

**Figure 1:**
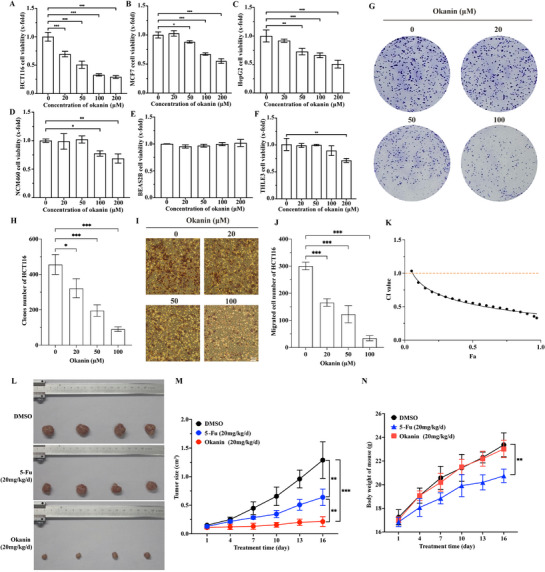
Okanin inhibits cancer cell growth in vitro and in vivo. A–C) Okanin inhibited the growth of colorectal (HCT116), breast (MCF7), and liver (HepG2) cancer cells. D–F) Okanin minimally affected viability of non‐cancerous colon (NCM460), liver (THLE3), and lung (BEAS2B) cells. G) Okanin suppressed clone formation in HCT116 cells. H) Quantification of clone numbers under increasing okanin concentrations. I) Okanin inhibited HCT116 migration (scale bar: 200 µm). J) Quantification of migrated cells under okanin treatment. K) Chou‐Talalay analysis revealed synergistic interactions (CI < 1) between okanin and 5‐Fu. L) Okanin inhibited colorectal cancer growth in nude mouse xenografts. M) Tumor volume measurements corresponding to (L). N) Okanin maintained body weight while 5‐Fu caused a significant reduction. ^*^: *p* < 0.05; ^**^: *p* < 0.01; ^***^: *p* < 0.001.

To determine whether okanin may have anti‐cancer activity, we performed MTT assay using various cancerous and non‐cancerous cells as experimental models. Okanin significantly inhibited the viability of HCT116 colorectal, MCF7 breast, and HepG2 liver cancer cells (**Figure** **1**A–C); in contrast, okanin had little or no effect on non‐cancerous NCM460 colorectal, THLE3 liver, and BEAS2B lung cells (Figure 1D–F). We then focused on the anti‐colorectal cancer effect, and found that okanin inhibits the clone formation (Figure 1G,H) and migration ability (Figure 1I,J) of HCT116. In addition, we examined whether the combinational use of okanin and 5‐fluorouracil (5‐Fu) could be therapeutically beneficial using Chou‐Talalay combination index analysis in HCT116 cells. As expected, okanin demonstrated strong synergy with 5‐Fu (CI < 1, Figure 1K). In the mouse xenograft model, okanin was able to inhibit substantially the in vivo growth of colorectal cancer cells (Figure 1L,M); impressively, under the experimental conditions, compared to 5‐Fu, okanin elicited a higher inhibition rate on cancer growth and much less reduction in animal body weight (Figure 1L–N). Histopathological analysis indicated that okanin did not cause detectable damage/toxicity in the major organs (i.e., liver and kidney). In contrast, 5‐Fu caused mesangial matrix deposition, mesangial expansion, and hepatic inflammatory (Figure S1A,B, Supporting Information). Clearly, under the experimental conditions, okanin potently inhibits the growth of the colorectal cancer cells without undesirable side effects.

### 2.2 PRDX5 is the Target of Okanin

**Figure 2:**
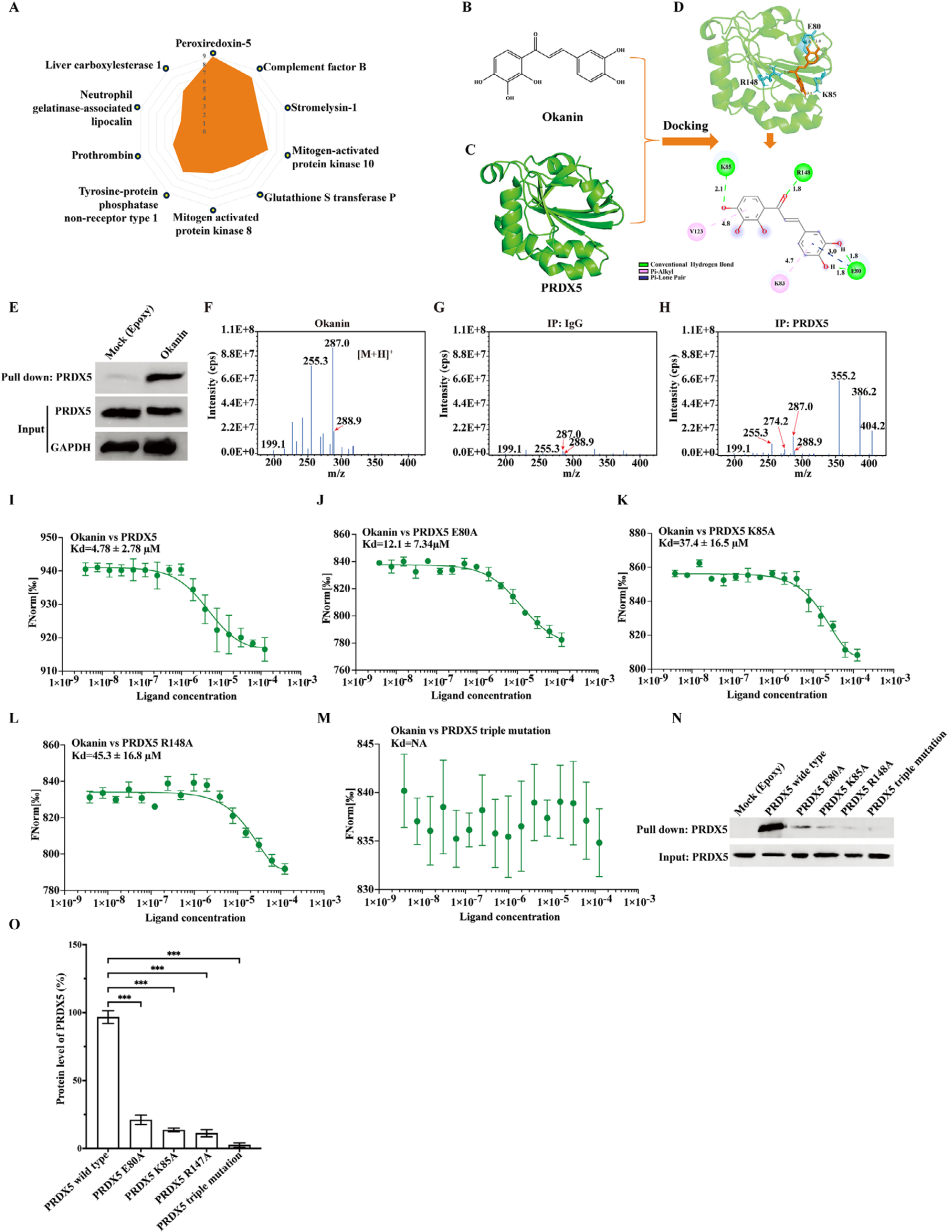
Okanin targets at PRDX5 in HCT116 cells. A) Top 10 candidate targets of PRDX5 with binding scores. B,C) Molecular structures of okanin (B) and PRDX5 (C). D) Molecular docking model showing okanin binding to PRDX5 residues E80, K85, and R148. E) Okanin pulldown of PRDX5. F–H) IP‐MS validation of okanin‐PRDX5 binding: characteristic okanin peaks (m/z 199.1, 255.3, 287.0, and 288.9) in free okanin (F), no specific binding in IgG control (G), and PRDX5‐bound okanin with diagnostic peptides enrichment (m/z 274.2, 355.2, 386.2, and 404.2) (H). I) Okanin‐PRDX5 binding affinity (Kd = 4.78 ± 2.78 µm). J–L) Weakened binding of okanin to PRDX5 mutants: E80A (Kd = 12.1 ± 7.34 µm), K85A (Kd = 37.4 ± 16.5 µm), and R148A (Kd = 45.3 ± 16.8 µm). M) Abolished okanin‐PRDX5 binding in triple mutant (E80A/K85A/R148A; MST). N) Disrupted binding by individual/triple mutants of PRDX5 confirmed by Western blot. O) Quantification of protein levels in (N). ^***^: *p* < 0.001.

To investigate the underlying mechanisms, we consulted the pharmMapper database for candidate targets of okanin (Table S1, Supporting Information). Amongst the top 10 proteins (**Figure** **2**A), PRDX5 had the highest score. In agreement with this, docking modeling upon the molecular structures of okanin (Figure 2B) and PRDX5 (Figure 2C) revealed that okanin's hydroxyl (‐OH) groups at the C3 and C4 of dihydroxyphenyl, as well as C4 positions of trihydroxyphenyl were critical for their binding (Figure 2D). Specifically, the C3 and C4 hydroxyls formed two hydrogen bonds (1.8 Å) with the carboxyl group of E80, while the C4 hydroxyl of trihydroxyphenyl interacted with the amine group of K85 via a hydrogen bond (2.1 Å). The carbonyl (C═O) group engaged in a hydrogen bond (1.8 Å) with the guanidinium group of R148 (Figure 2D). Moreover, okanin's aromatic ring participated in pi‐alkyl interactions (purple lines) with the aliphatic side chains of K85 (4.7 Å) and V123 (4.8 Å), and a pi‐lone pair interaction (blue line, 3.0 Å) with the carbonyl oxygen of E80 (Figure 2D). These interactions stabilized the binding of okanin to PRDX5's surface groove. Molecular dynamics simulation showed that okanin‐PRDX5 complexes were structurally stable (root mean square deviation, RMSD < 0.5 nm over 50 ns; Figure S1C, Supporting Information). Indeed, in the subsequent experiments, the binding between okanin and PRDX5 was demonstrated. The results from the epoxy‐activated sepharose 6B‐Hp II pull‐down assay showed that okanin‐beads pulled down RPDX5 (Figure 2E), but not other members of PRDX family (PRDX1 to PRDX4 and PRDX6; Figure S1D, Supporting Information). Critically, IP‐MS analysis revealed a unique binding signature in the PRDX5‐okanin complex: while free okanin showed characteristic peaks at m/z 199.1, 255.3, and 287.0 (base peak intensity: 1.1E8 cps), the PRDX5‐IP group exhibited these characteristic okanin peaks alongside with prominent novel peaks at m/z 274.2, 355.2, 386.2, 404.2 which corresponded to the characteristic peptide fragments of PRDX5 (Figure S1E, Supporting Information); in contrast, IgG controls showed only minimal okanin peaks (non‐specific background) without the PRDX5 signature, confirming the target‐specific binding (Figure 2F–H). In MST assay, okanin and PRDX5 were found to bind each other with Kd = 4.78±2.78 µm (Figure 2I). In experiments using proteins mutated at E80A, K85A and R148A alone or in combination (Figure S1F–H, Supporting Information), we observed that mutation at any of the amino acid residues reduced the binding between okanin and PRDX5, that the Kd value was 12.1 ± 7.34, 37.4 ± 16.5, and 45.3 ± 16.8 µm, respectively (Figure 2J–L). Okanin loss of binding ability with PRDX5 when the three residues were simultaneously mutated (Figure 2M). Okanin‐beads pull down results also indicated that it nearly completely disrupted the binding if simultaneous mutation at the three residues (Figure 2N,O). Collectively, these results indicate that PRDX5 is the target of okanin, and the binding between okanin and PRDX5 is mediated by residues E80, K85, and R148.

### 2.3 Okanin Suppresses the Peroxidase Activity of PRDX5 in a Cell‐Free Assay

**Figure 3:**
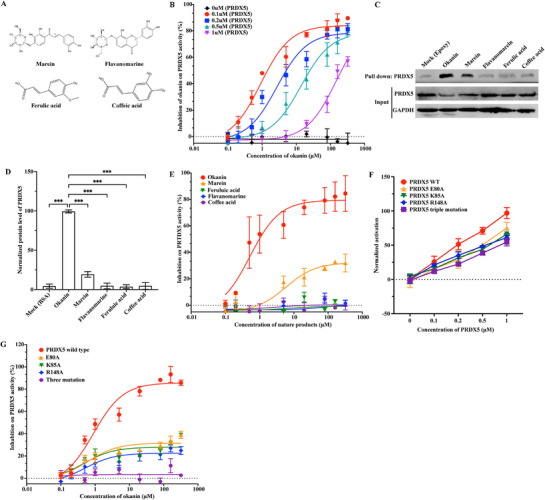
Okanin inhibits the enzymatic activity of PRDX5 specifically. A) Molecular structures of marein, flavanomarein, ferulic acid, and caffeic acid. B) Concentration‐dependent inhibition of PRDX5 peroxidase activity by okanin. C) Marein exhibited partial (≈20%) PRDX5 binding capacity versus okanin; flavanomarein, ferulic acid, and caffeic acid showed negligible binding. D) Quantification of protein levels in (C). E) Inhibition rates for PRDX5 peroxidase activity: okanin (85%), marein (23%); flavanomarein, ferulic acid, and caffeic acid showed no detectable inhibition. F) Compared to wild‐type PRDX5, mutant PRDX5 proteins (E80A, K85A, R148A, and triple mutant E80A/K85A/R148A) exhibit reduced basal peroxidase activity. G) Compared to wild‐type PRDX5, mutant PRDX5 proteins exhibit significantly reduced okanin‐induced inhibition of peroxidase activity. ^***^: *p* < 0.001.

Next, we moved to test whether okanin inhibited the peroxidase activity of PRDX5 and the specificity of the inhibition; the latter was assessed by comparisons of inhibitory activity between okanin and similar (marein and flavanomarine) or different (ferulic and caffeic acid) chemical structures. Okanin reduces oxidative stress via oxidation of sulfhydryl groups to form disulfide bonds that can be reduced back to sulfhydryl groups by thioredoxin‐1^[^ ^17^ ^]^ (Figure S2A, Supporting Information). Its structure features a planar α, β‐unsaturated ketone scaffold connecting two hydroxyl‐rich aromatic rings: the A ring (2,3,4‐trihydroxyphenyl) and B ring (3′,4′‐dihydroxyphenyl, catechol), enabling hydrogen, Pi‐Alkyl, and Pi‐Lone Pair bonding with PRDX5 (Figure 2D). In contrast, marein modifies the A ring by replacing the 4‐OH with a bulky glucose moiety, which may sterically hinder access to this hydroxyl. Flavanomarein has a similar B‐ring catechol compared to okanin, but has no α, β‐unsaturated ketone scaffold and glycosylates the A‐ring 7‐OH, altering planarity and hydroxyl availability. Ferulic acid and caffeic acid though sharing phenolic hydroxyls with okanin, lack the chalcone scaffold entirely (**Figure** **3**A). In our experiments, okanin inhibited the peroxidase activity of PRDX5 in a concentration‐dependent manner (Figure 3B). Marein displayed ≈20% of binding efficacy but failed to maintain structural integrity (RMSD > 0.5 nm at 20 ns; > 2.0 nm at 40 ns MD simulations; Figure S2B, Supporting Information), while flavanomarine, ferulic, and caffeic acid exhibited basically no binding potential (Figure 3C,D). Consistently, marein showed ≈20% inhibition rate, while the other compounds had no detectable inhibitory effect on the activity of PRDX5 (Figure 3E). Moreover, mutations at E80A, K85A, R148A, or the combined three reduced the basal enzymatic activity of PRDX5 by 20%, 25%, 28%, and 40%, respectively (Figure 3F). The mutations coonfered resistant to the inhibition effect of okanin on the enzymatic activity of PRDX5 (80%, 85%, 88%, and 100%, respectively; Figure 3G). It appears that the binding between okanin and PRDX5 shows a certain level of specificity, likely involving the three amino acid residues in PRDX5, which results in the inhibition of PRDX5 peroxidase activity.

### 2.4 Okanin Triggers the Ubiquitination Degradation of PRDX5

**Figure 4:**
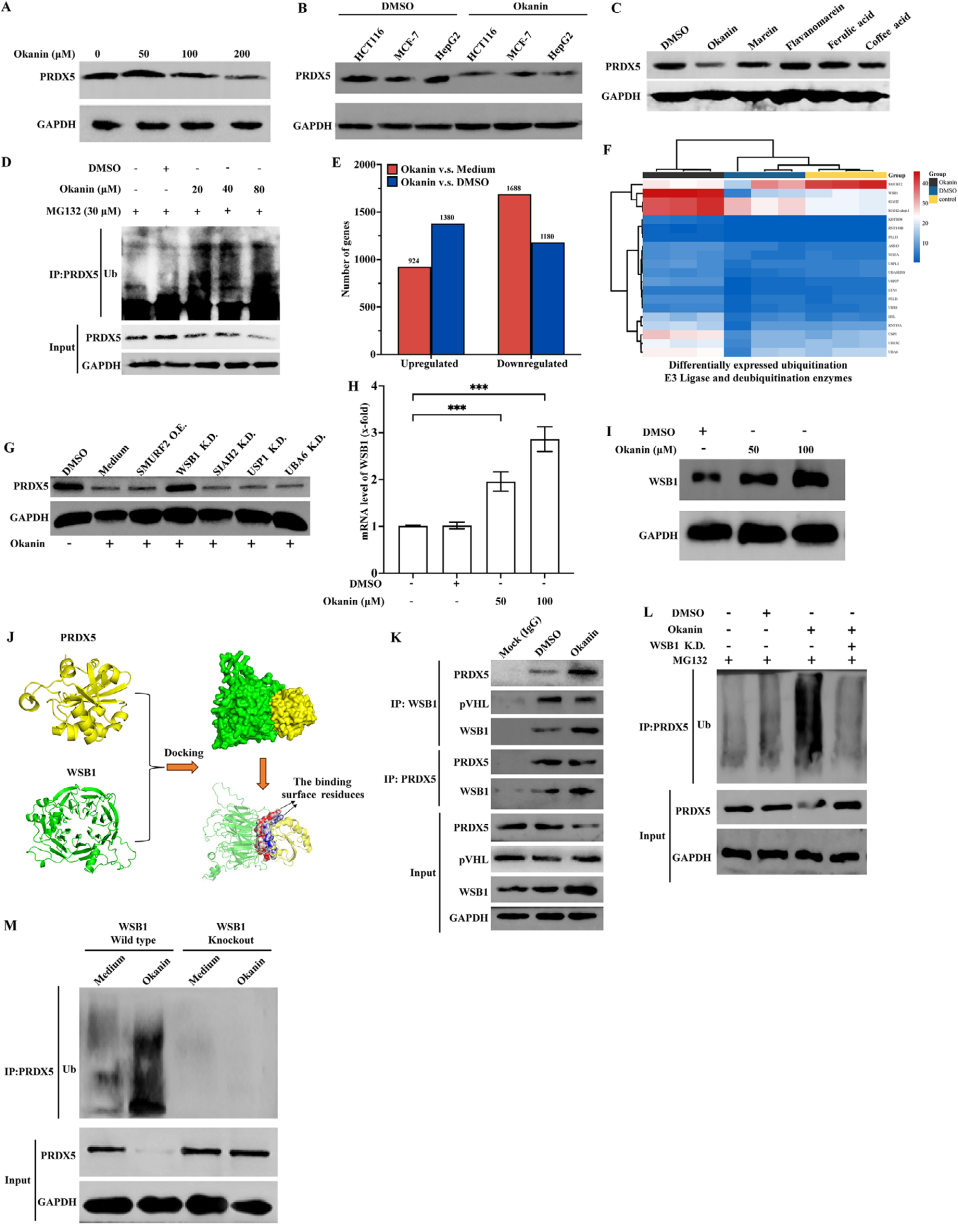
)Okanin triggers PRDX5 ubiquitination degradation. A) Concentration‐dependent decrease in PRDX5 protein in HCT116 cells with okanin treatment. B) PRDX5 reduction in multiple cancer lines (HCT116, MCF7, HepG2). C) Marein induced minor PRDX5 reduction (≈20%); flavanomarein, ferulic acid, and caffeic acid showed no effect. D) Concentration‐dependent increase in PRDX5 ubiquitination. E) Differentially expressed genes: okanin versus medium (924 up/1380 down) and okanin versus DMSO (1688 up/1180 down). F) Top 20 dysregulated E3 ligases/deubiquitinases. G) WSB1 knockdown rescued okanin‐induced PRDX5 degradation. H,I) Okanin upregulated WSB1 mRNA and protein. J) Docking model of WSB1‐PRDX5 interaction. K) Co‐IP confirmed WSB1‐PRDX5 binding (IgG negative control; pVHL positive control). L) WSB1 knockdown attenuated okanin‐induced PRDX5 ubiquitination. M) WSB1 knockout abolished okanin‐induced PRDX5 ubiquitination. ^***^: *p* < 0.001.

We also wondered whether okanin could alter the protein spebility/level of PRDX5, Indeed, okanin reduced the protein level of PRDX5 in a concentration‐dependent manner not only in HCT116 (**Figure** **4**A; Figure S3A, Supporting Information) and SW480 colorectal cancer cells (Figure S3B, Supporting Information), but also in MCF7 breast cancer and HepG2 liver cancer cells (Figure 4B; Figure S3C, Supporting Information), without altering the protein levels of other PRDX family members (Figure S3D,E, Supporting Information). In contrast, flavanomarine, ferulic, and caffeic acid had no such effect, except that marein, which showed limited structural similarity with okanin reduced the GRPDX5 protein level by ≈20% (Figure 4C; Figure S3F, Supporting Information). To address the question of how the protein level was decreased, we found that okanin increased the ubiquitination of PRDX5 in a concentration‐dependent manner in HCT116 cells (Figure 4D). Then, we asked how the ubiquitination occurred. Transcriptomic profiling revealed that 924 and 1380 genes were upregulated and downregulated by okanin, respectively, compared to PBS, and 1688 and 1180 genes were upregulated and downregulated, respectively, compared to the solvent DMSO (Figure 4E). The top 20 differentially expressed E3 ubiquitin‐protein ligase and deubiquitination enzymes genes are shown in Figure 4F, amongst which the top 5 were subjected to further functional characterization by overexpression or knockdown (Figure S3G–K, Supporting Information). The results showed that only WSB1 played a role, as knockdown of WSB1, but not others, blocked the okanin‐decreased PRDX5 protein level (Figure 4G; Figure S3L, Supporting Information). Okanin concentration‐dependently upregulated WSB1 expression at both of mRNA and protein levels, with orthogonal validation in both HCT116 (mRNA: Figure 4H; protein: Figure 4I; Figure S3M, Supporting Information) and SW480 cells (mRNA: Figure S3N; protein: Figure S3O, Supporting Information). Molecular docking analyses suggested that WSB1 and PRDX5 were able to bind to each other (Figure 4J). The binding interface between WSB1 and PRDX5 involves 37 and 29 mino acid residues on WSB1 and PRDX5, respectively (interface area = 1046.3 Å^2^ and Δ^i^G = −15.4 kcal mol^−1^), stabilized by nine hydrogen bonds (Figure S4A, Supporting Information). Their binding was supported by Co‐IP experiments, while okanin enhanced the binding between WSB1 and PRDX5 (Figure 4K) without direct binding with WSB1 (Figure S3P, Supporting Information). Importantly, the binding concomitantly occurred with PRDX5 ubiquitination. Knocking down of WSB1 significantly attenuated the ubiquitination of PRDX5 by okanin treatment (Figure 4L). Furthermore, genetic knockout of WSB1 completely abolished okanin‐induced ubiquitination and subsequent degradation of PRDX5 (Figure 4M; Figure S3Q, Supporting Information). In contrast, WSB1 overexpression enhanced the ubiquitination (Figure S3R, Supporting Information). WSB1 knockdown reversed the okanin‐decreased PRDX5 without modifying the protein levels of PRDX1‐4 and PRDX6 (Figure S3D,E, Supporting Information), suggesting that WSB1 specifically mediates the PRDX5 ubiquitination degradation, resulting in the drop of PRDX5 protein level.

### 2.5 Actions of Okanin at PRDX5 Triggers Apoptosis and Ferroptosis Independently

**Figure 5:**
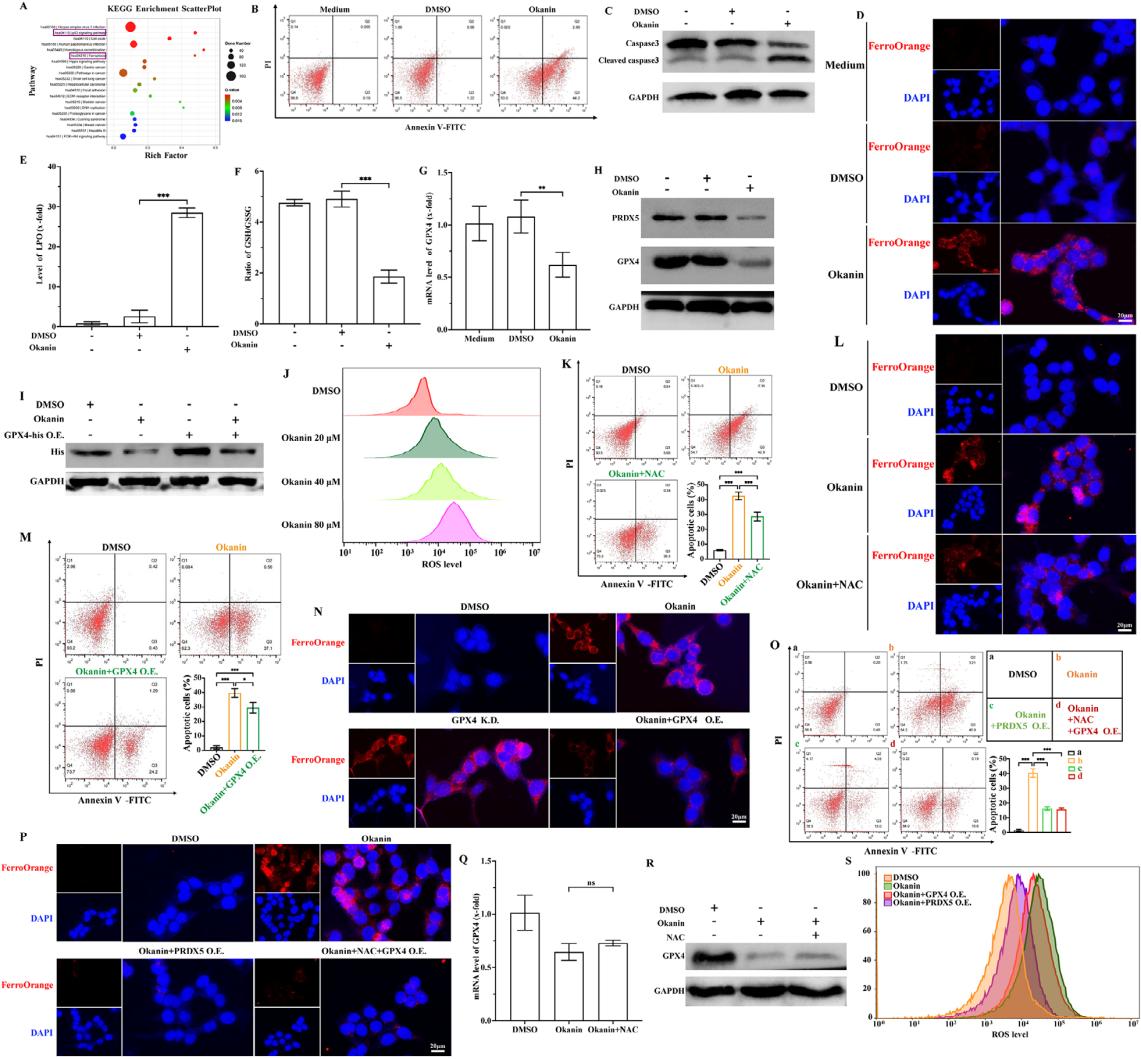
Okanin triggers apoptosis and ferroptosis of colorectal cancer cells through PRDX5. A) KEGG enrichment of differentially expressed genes in apoptosis and ferroptosis pathways. B,C) Okanin induced apoptosis: Annexin V‐FITC/PI staining (B) and caspase‐3 cleavage (C). D,E) Okanin increased cytoplasm free Fe^2+^ accumulation (D) and LPO production (E). F) Okanin decreased the ratio of GSH/GSSG. G,H) Okanin inhibited GPX4 expression in both mRNA (G) and protein (H) levels. I) Okanin also reduced exogenous GPX4 protein level. J) Okanin concentration‐dependently promoted ROS production. K,L) NAC attenuates okanin‐induced apoptosis (annexin V‐FITC/PI staining; K) and ferroptosis (free Fe^2+^ staining; L). M,N) GPX4 overexpression inhibited apoptosis (annexin V‐FITC/PI staining; M) and ferroptosis (free Fe^2+^ staining; N). O,P) Combined NAC and GPX4 overexpression blocked both apoptosis (annexin V‐FITC/PI staining; O) and ferroptosis (free Fe^2+^ staining; P), like PRDX5 overexpression. Q,R) NAC did not alter expression of GPX4 at mRNA (Q) and protein (R) levels. S) PRDX5 (not GPX4) overexpression inhibited okanin‐induced ROS production. The scale bar is 20 µm (fluorescence imaging). ^**^: *p* < 0.01; ^***^: *p* < 0.001.

Then, a key question was whether the inhibition of PRDX5 modified the cell fate/viability. Our transcriptomic profiling and KEGG annotation results (Table S2, Supporting Information) revealed many differentially expressed genes that could be clustered into apoptosis and ferroptosis signaling pathways (**Figure** **5**A). Experimentally, okanin did induce apoptosis evidenced by the increase in of sub‐G1 cells and the cleavage of caspase‐3 (Figure 5B,C) as well as ferroptosis evidenced by increment in free Fe^2+^ (Figure 5D; Figure S5A, Supporting Information) and lipid peroxidation (LPO) (Figure 5E; Figure S5B, Supporting Information) with a concomitant decrease in glutathione (GSH)/glutathione disulfide (GSSG) ratio (Figure 5F) and lipid peroxide GPX4 (Figure 5G–I; Figure S5C, Supporting Information) in HCT116 cells and, similalry, in SW480 cells (Figure S5D–F, Supporting Information).

Alongside the cell death, we found that okanin or PRDX5 knockdown (Figure S5G, Supporting Information) caused ROS production in a concentration‐dependent manner (Figure 5J). In contrast, PRDX5 overexpression reduced the ROS level, irrespective to the present or absence of okanin (Figure S5H,I, Supporting Information); N‐acetyl cysteine (NAC) as ROS reducer markedly inhibited apoptosis (Figure 5K) as well as slightly but yet significantly attenuated ferroptosis (Figure 5L; Figure S5J,K, Supporting Information). Moreover, okanin decreased the protein level of GPX4 (Figure 5G–I; Figure S5A, Supporting Information), and its overexpression attenuated the apoptosis (Figure 5M; Figure S5L, Supporting Information) and largely inhibited the ferroptosis (Figure 5N; Figure S5M,N, Supporting Information). Importantly, the combination of NAC and GPX4 overexpression, as well as PRDX5 overexpression, nearly completely blocked the apoptosis (Figure 5O) and ferroptosis (Figure 5P; Figure S5O,P, Supporting Information). To investigate the relationship between NAC and GPX4, we found that NAC did not significantly alter the decrease in GPX4 by okanin treatment, either at the mRNA (Figure 5Q) or protein level (Figure 5R), and GPX4 overexpression did not significantly modify the ROS production (Figure 5S). Taken together, the results suggest both ROS and GPX4 are downstream of PRDX5 and serve as independent signaling pathways of apoptosis and ferroptosis. It appears that ROS signals predominantly for apoptosis, while GPX4 is more for ferroptosis.

### 2.6 Apoptosis and Ferroptosis are Independent Cell Death Events

**Figure 6:**
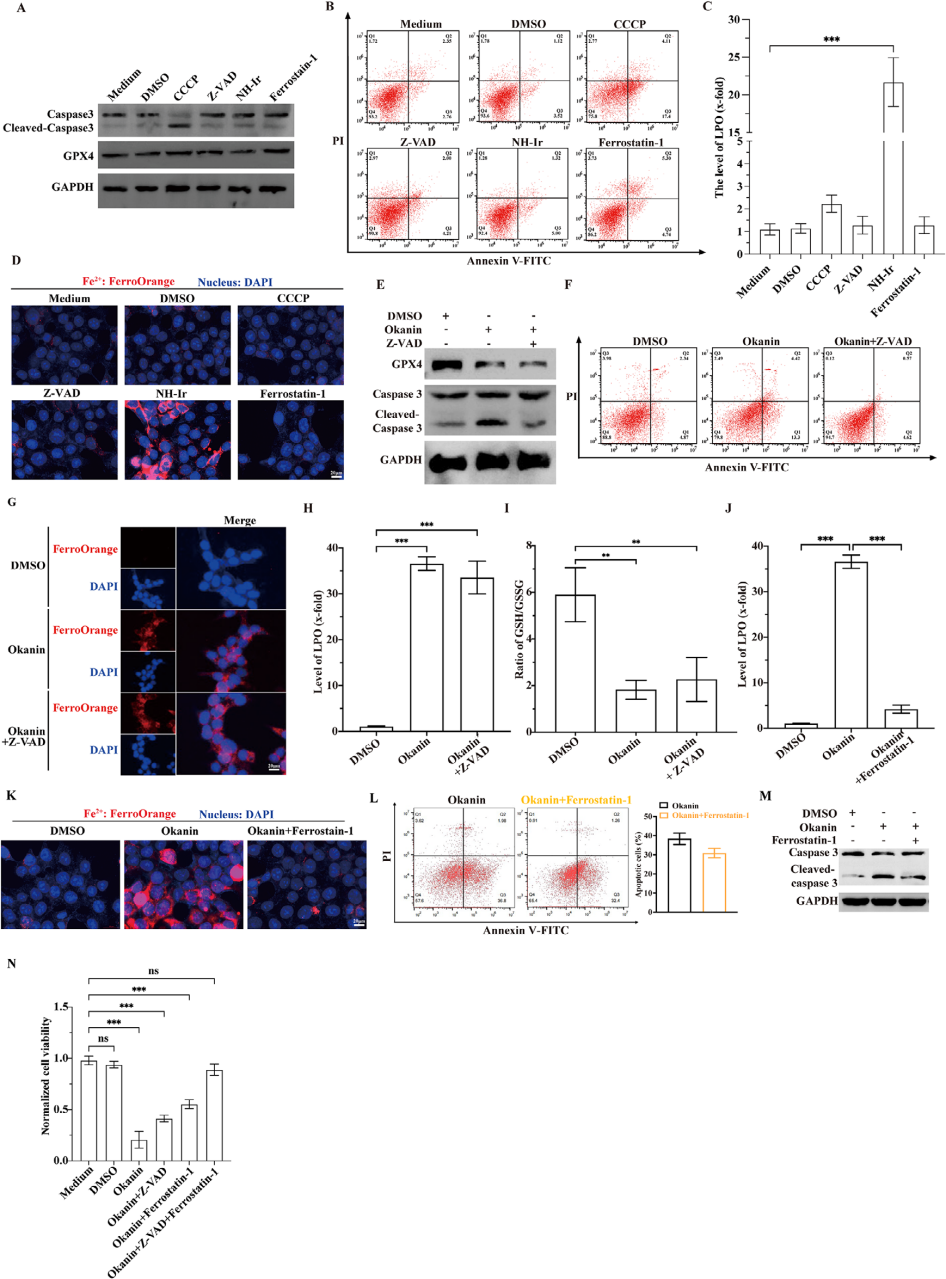
Okanin‐triggered apoptosis and ferroptosis are independent cell death events. A,B) Apoptosis was validated through caspase‐3 cleavage (A) and Annexin V‐FITC/PI dual staining (B) under modulation by apoptosis inducers (CCCP, 10 µm) or inhibitors (Z‐VAD‐FMK, 20 µm), and ferroptosis inducer (NH‐Ir, 5 µm) or inhibitor (Ferrostatin‐1, 1 µm). C,D) Ferroptosis was also assessed with both apoptosis and ferroptosis modulators treatment by LPO (C) and free Fe^2+^ (D) levels analysis. E–I) Pan‐caspase inhibitor Z‐VAD‐FMK (20 µM) selectively blocked apoptotic markers, including caspase‐3 activation (E) and Annexin V⁺ cells (F), without altering ferroptotic signatures: GPX4 suppression (E), free Fe^2+^ accumulation (G), LPO elevation (H), or GSH/GSSG ratio reduction (I). J–M) Conversely, ferrostatin‐1 (1 µm) inhibited ferroptotic features (LPO in J; free Fe^2+^ overload in K) while sparing apoptotic indices (Annexin V⁺ cells population in L; caspase‐3 cleavage in M). N) Combined Z‐VAD‐FMK + Ferrostatin‐1 synergistically restored the viability of okanin‐treated cells. The scale bar is 20 µm (fluorescence imaging). ^**^: *p* < 0.01; ^***^: *p* < 0.001.

Given that okanin triggered both apoptosis and ferroptosis, we wondered whether the two cell death processes might be related to each other. To dissect their interplay, HCT116 cells were co‐treated with okanin and either apoptosis modulators (carbonyl cyanide m‐chlorophenyl hydrazone, CCCP as an inducer or benzyloxycarbonyl‐Val‐Ala‐Asp(OMe)‐fluoromethylketone, Z‐VAD‐FMK as an inhibitor) or ferroptosis modulators (NH‐Ir as an inducer or Ferrostatin‐1 as an inhibitor). When used alone, apoptosis and ferrotopsis modulators had no effects on ferroptosis (GPX4 suppression, LPO and Fe^2^⁺ accumulation) and apoptosis (caspase‐3 cleavage and Annexin V positively staining), respectively, irrespective to the presence or absence of okanin (**Figure** **6**A–D). Importantly, apoptosis inhibitor (Z‐VAD‐FMK) specifically blocked okanin‐induced apoptosis (Figure 6E,F) without affecting ferroptosis (Figure 6G–I), and ferroptosis inhibitor (Ferrostatin‐1) specifically blocked okanin‐induced ferroptosis (Figure 6J,K) without altering apoptosis (Figure 6L,M). Moreover, the combined use of both inhibitors reversed the inhibitory effect of okanin on cell viability (Figure 6N). Therefore, the apoptosis and ferroptosis are likely to be independent cell death events during okanin treatment.

### 2.7 Okanin‐Decreased GPX4 Protein Level is Attributed to SIAH2‐Mediated Ubiquitination Degradation

**Figure 7:**
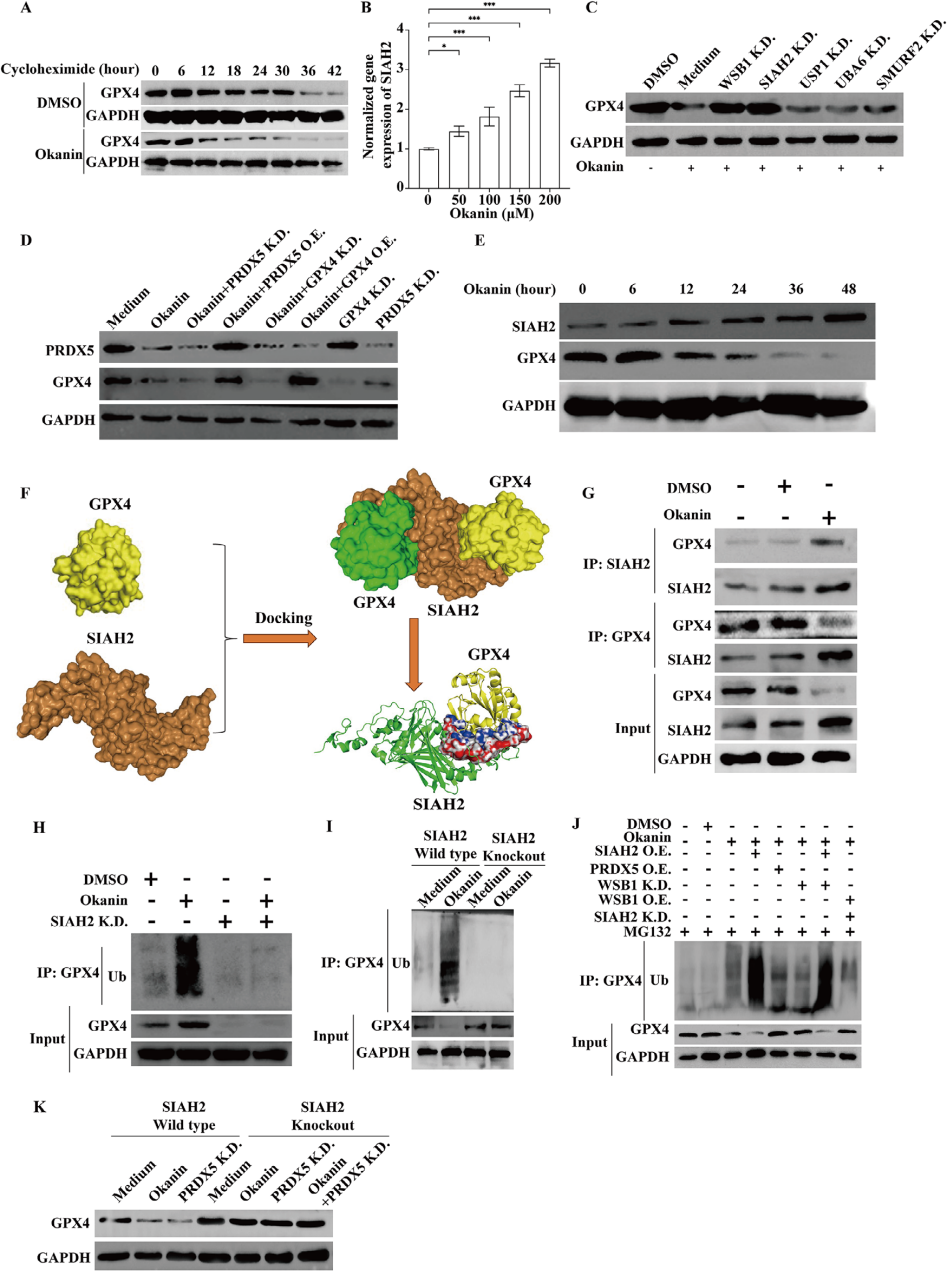
SIAH2 mediates GPX4 ubiquitination. A) Okanin significantly shortened the half‐life time of GPX4. B) Okanin concentration‐dependently upregulated the mRNA level of SIAH2. C) SIAH2 or WSB1 knockdown reversed okanin‐decreased protein level of GPX4. D) PRDX5 knockdown or overexpression altered okanin‐mediated GPX4 changes, while GPX4 modulation does not affect PRDX5. E) Time‐dependent negative correlation between SIAH2 and GPX4 protein levels under okanin treatment. F) Molecular docking model of SIAH2 and GPX4. G) Okanin strengthened GPX4‐SIAH2 binding, as evidenced by reciprocal co‐immunoprecipitation. H) SIAH2 knockdown inhibited okanin‐promoted ubiquitination of GPX4. I) SIAH2 knock out abolished okanin‐induced ubiquitination of GPX4. J) SIAH2 overexpression promotes GPX4 ubiquitination despite PRDX5 overexpression or WSB1 knockdown. K) Neither okanin treatment nor PRDX5 inhibition reduced GPX4 protein level in SIAH2 knockout cells, whether administered alone or in combination.

Our next question was how okanin downregulated GPX4 (Figure 5G–I), which can be regulated at both transcription and protein stability levels.^[^ ^11^, ^31^ ^]^ Our results showed that the half‐life of GPX4 was shortened by okanin in the presence of cyclohexamide, which inhibits protein translation (**Figure** **7**A; Figure S6A, Supporting Information). To examine whether reduced protein stability was due to ubiquitination degradation, we searched for its E3 ligase from the top 5 differentially expressed ligase genes, which included the upregulation of SIAH2 (Figure 4F). Indeed, okanin upregulated the expression of SIAH2 in a concentration dependent manner (Figure 7B; Figure S3O, Supporting Information). Knockdown of SIAH2 or WSB1 counteracted the downregulation of GPX4 (Figure 7C; Figure S6B, Supporting Information), while the other three okanin responsive ligases had no such functional roles (Figure 7C). Since WSB1 mediated PRDX5 ubiquitination, we are curious about the relationship between PRDX5 and GPX4. Interestingly, overexpression and knockdown of PRDX5 promoted and suppressed GPX4 expression, respectively, under okanin treatment (Figure 7D; Figure S6C, Supporting Information). In contrast, neither GPX4 knockdown nor overexpression altered okanin‐mediated changes in PRDX5 level (Figure 7D; Figure S6C, Supporting Information), indicating that PRDX5 acts upstream of GPX4 in the pathway. Moreover, the following experimental data, together with the above observations, supported the view that SIAH2 was the ubiquitination ligase for GPX4: 1) upon okanin treatment, a time‐dependent increase in SIAH2 correlated with the decrease in GPX4 (Figure 7E; Figure S6D, Supporting Information); 2) Structural modeling revealed a high‐affinity between SIAH2 and GPX4 (Figure 7F), which form a complex (Δ^i^G = −19.3 kcal mol^−1^) with an extensive binding interface (1240.3 Å^2^) involving 46 SIAH2 residues and 31 GPX4 residues, stabilized by 14 hydrogen bonds as visualized in Figure S4B (Supporting Information); 3) co‐IP assay revealed a basal binding between SIAH2 and GPX4, which was greatly enhanced by okanin that upregulated SIAH2 (Figure 7G); 4) okanin increased the ubiquitination of GPX4, which was attenuated by SIAH2 knockdown (Figure 7H) and abolished by SIAH2 knockout (Figure 7I; Figure S6E, Supporting Information). In this group of experiments, we also found that PRDX5 overexpression or WSB1 knockdown counteracted the GPX4 ubiquitination, which was inhibited when SIAH2 was overexpressed (Figure 7J). Crucially, GPX4 levels remained stable even under PRDX5 suppression in SIAH2 knockout cells, confirming SIAH2 is indispensable for this pathway (Figure 7K). Collectively, these results suggest that okanin decreases the level of GPX4 by reducing transcription and triggering SIAH2‐medaited ubiquitination degradation; the latter is downstream of PRDX5.

### 2.8 Okanin Targets at PRDX5 to Inhibit Cancer Cell Growth In Vivo

**Figure 8:**
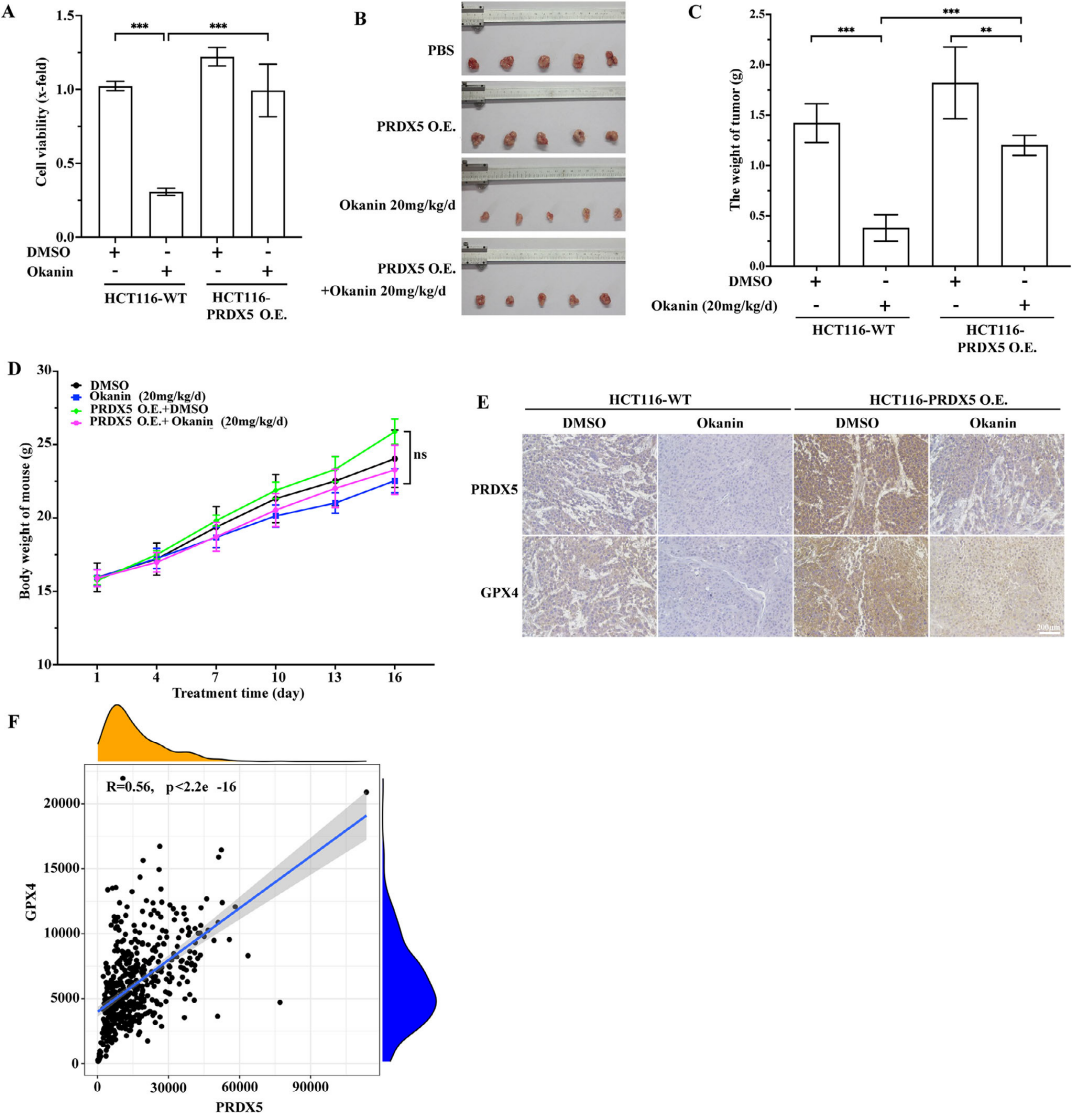
PRDX5 overexpression inhibits okanin‐promoted cancer cell growth in vivo. A) PRDX5 stable overexpression rescued okanin‐inhibited cell growth of HCT116 in vitro. B) Corresponding rescue of tumor growth in nude mouse xenografts. C) Quantification of tumor weights from (B). D) Okanin maintains body weight across groups (n = 5 mice/group). E) Okanin reduced PRDX5 and GPX4 protein expression in tumor tissues by immunohistochemistry. F) Positive correlation between PRDX5 and GPX4 expression in colorectal adenocarcinoma patient samples. Scale bar: 200 µm (immunohistochemistry imaging). ^**^: *p* < 0.01; ^***^: *p* < 0.001.

Finally, we sought to assess whether okanin targets at PRDX5 to inhibit cancer cell growth in nude mice model, for which we generated a stable PRDX5‐overexpressing cell line, hereby designated HCT116‐PRDX5 O.E. and the wild type cells as HCT116‐WT (Figure S7A, Supporting Information). At a concentration of 100 µg mL^−1^ in 24 h, okanin reduced the 75% and 10% of viability of HCT116‐WT and HCT116‐PRDX5 O.E., respectively; the latter was not significantly different from that in the control group (**Figure** **8**A). Likewise, in xenograft mice model, okanin at the dose of 20 mg kg day^−1^ reduced the growth of HCT116‐WT and HCT116‐PRDX5 O.E. cells by 70% and 20%, respectively (Figure 8B,C). The average body weight remained comparable amongst all of the experimental groups (Figure 8D). In mice inoculated with either HCT116‐WT or HCT116‐PRDX5 O.E. cells, okanin injection i.p. downregulated the protein levels of both PRDX5 and GPX4 in all tumor tissues (Figure 8E). However, even upon okanin treatment, PRDX5 and GPX4 levels remained significantly higher in tumors of HCT116‐PRDX5 O.E. cells than that in tumors of HCT116‐WT counterparts (Figure 8E). We were unable to quantify the ROS level in the tumor tissues due to the lack of fresh samples. In the bioinformatics era, information retrieved from of TCGA database indicated that expression of PRDX5 and GPX4 were increased in various blood and solid cancers (Figure S7B,C, Supporting Information). There was a positive correlation between PRDX5 and GPX4 levels in colorectal adenocarcinoma patients (Figure 8F).

## 3 Discussion

Due to persistent challenges with drug resistance cancer and dose limiting effect, the identification of new therapeutic agents and strategies is a focus of extensive ongoing efforts in cancer therapy. We found that okanin in vitro and in vivo inhibited the growth of colorectal cancer cells by directly targeting at PRDX5 to trigger both apoptosis and ferroptosis, suggesting that PRDX5 is a target of cancer chemotherapy. It is possible that alteration of PRDX5 can benefit cancer management from other angles; knockdown of PRDX5 sensitizes non‐small cell lung cancer cells to cisplatin, as well as inhibits cancer cell growth in zebrafish model.^[^ ^32^ ^]^

We demonstrated that okanin directly bound and suppressed PRDX5 via two mechanisms: inhibition of enzymatic activity and induction of protein ubiquitination degradation, despite that we were unable to assess their relative contributions to the functional alteration upon treatment. The catalytic domain of PRDX5 contains two cysteine residues (Cys 48 and Cys 152), the Cys48 thiol (Cys48‐SH) is oxidized to sulfenic acid (Cys48‐SOH) during scavenging radicals, after which Cys152‐SH reacts with Cys48‐SOH to form an intramolecular disulfide bond,^[^ ^33^ ^]^ which is recovered into two thiol groups by TXN. Inhibitors of PRDX family members usually inhibit PRDX enzymatic activity by binding to the catalytic domain; for example, CoAlation inhibits to PRDX1 by covalently binding to Cys48.^[^ ^34^ ^]^ Differently, in the present study, okanin bound to PRDX5 via interaction with three residues (E80, R148, and K85) (Figure 2B–H) that were located in a region opposite to the catalytic domain of C48 and C152 (Figure 2B), despite that it resulted in the same functional consequence of activity inhibition. Although molecular dynamics simulation revealed a conformational shift in PRDX5's thioredoxin fold after okanin binding (Figure S7D, Supporting Information), there is a lack of direct evidence. Presumably, the binding alters the protein configuration and thus hinders the transient conformational integration of Cys48‐SH and Cys152‐SH, which disrupts the formation of catalytic domain(s).^[^ ^34^ ^]^ PRDX activity was also tightly controlled at the protein expression level by post‐translational modifications, as was shown in the study that okanin decreased its protein level via WSB1‐mediated ubinquitination degradation (Figure 4L). Other mechanisms of PRDX5 regulation include reversible thiol modifications by S‐sulphenylation, S‐sulphinylation, S‐glutathionylation, S‐nitrosylation, phosphorylation, and acetylation.^[^ ^16^, ^35^ ^]^ There is a report that WSB1 appears to be an oncogene that increases cancer development and thus results in poor prognosis.^[^ ^21^ ^]^ This discrepancy could potentially be explained by differences in experimental conditions, such as different cell models used.

Our findings showed okanin‐decreased PRDX5 led to apoptosis and ferroptosis, which is supported by the experimental knockdown of PRDX5 leading to oxidative stress and apoptosis in A549 cells.^[^ ^36^ ^]^ Apoptosis and ferroptosis can be independent^[^ ^37^ ^]^ or related in an inconsequential manner.^[^ ^38^ ^]^ In the study, there was no evidence of inter‐talk between apoptosis and ferroptosis, despite that they shared the same mediators, ROS and GPX4. It is possible that ROS and GPX4 are shared upstream signals of two pathways that predominantly lead to apoptosis and ferroptosis, respectively. This is in agreement with reports that altering the levels of ROS or GPX4 is linked to apoptosis and ferroptosis.^[^ ^39^ ^]^

Relationship between PRDX5 and GPX4 remains unknown. Our results suggest that downregulation of PRDX5 not only inhibits the transcription of GPX4 (Figure 5G) but also leads to ubiquitination degradation of GPX4 via the E3 ligase SIAH2 (Figure 7D,G), which establishes a signaling link between the two molecules. Mechanistically, we suspect that the effects of okanin on PRDX5 disrupt its binding^[^ ^18^ ^]^ with nuclear factor erythroid 2–related factor 2 (NRF2), and SIAH2 that can bind to NRF2^[^ ^40^ ^]^ might cause NRF2 ubiquitination degradation, both of which can compromise transcription of GPX4 by NRF2 as a transcription factor.^[^ ^41^ ^]^ Under the experimental conditions, Signaling of ROS and GPX4 that were downstream of PRDX5 let predominantly to apoptosis and ferroptosis, respectively (Figure 5A–H), while overexpression of PRDX5 nearly block both cell death events, which supports the key controlling role of PRDX5 in the cell deaths and thus the importance of PRDX5 as the okanin direct target and cell signaling mediator.

Ubiquitination plays a significant role in regulating apoptosis and ferroptosis.^[^ ^42^, ^43^ ^]^ A variety of pro‐ or anti‐apoptosis molecules, such as p53, BID, Bax, Bak, Bim, Bcl‐2, Bcl‐xL, and Mcl‐1,^[^ ^43^ ^]^ are subjected to ubiquitination modification that modifies their functional roles in determining cell fate. For example, pro‐apoptotic Bim can be regulated by several E3 ligases, including Cbl, TRIM2, and CRL2CIS complex.^[^ ^43^ ^]^ Anti‐apoptotic Mcl‐1 can be ubiquitination degraded by E3 ligase TRIM17 and stabilized by deubiquitinating enzyme USP9X or USP13.^[^ ^43^ ^]^ Pro‐ferroptosis NRF2 can underdo ubiquitination by the E3 ligases kelch‐like ECH‐associated protein 1^[^ ^44^ ^]^ and deubiquitination by ubiquitin specific peptidase 11,^[^ ^45^ ^]^ while anti‐ ferroptosis solute carrier family 7 member 11, can be regulated by the E3 ligases tripartite motif containing 26^[^ ^46^ ^]^ and OTUB1.^[^ ^47^ ^]^ Apparently, an E3 ligase has different protein targets, likewise, a protein can be modified by different ligase.^[^ ^48^ ^]^ This explains why, in the study, actions of WBS1 and SIAH2 inhibit cancer cell growth, which is different from other observations that they favor tumorigenesis and tumor progression.^[^ ^24^ ^]^

Despite that, our findings highlight okanin's therapeutic potential, the application value remains to be validated. The chronic toxicity profile, particularly in PRDX5‐rich tissues like liver and kidney, and potential off‐target effects stemming from okanin's flavonoid scaffold need systematic evaluation. The current preclinical model may not fully mirror the complexity of human tumor microenvironments where PRDX5 engages dynamic redox networks. Clearly, clinical trials will address all concerns.

## 4 Conclusion

**Figure 9:**
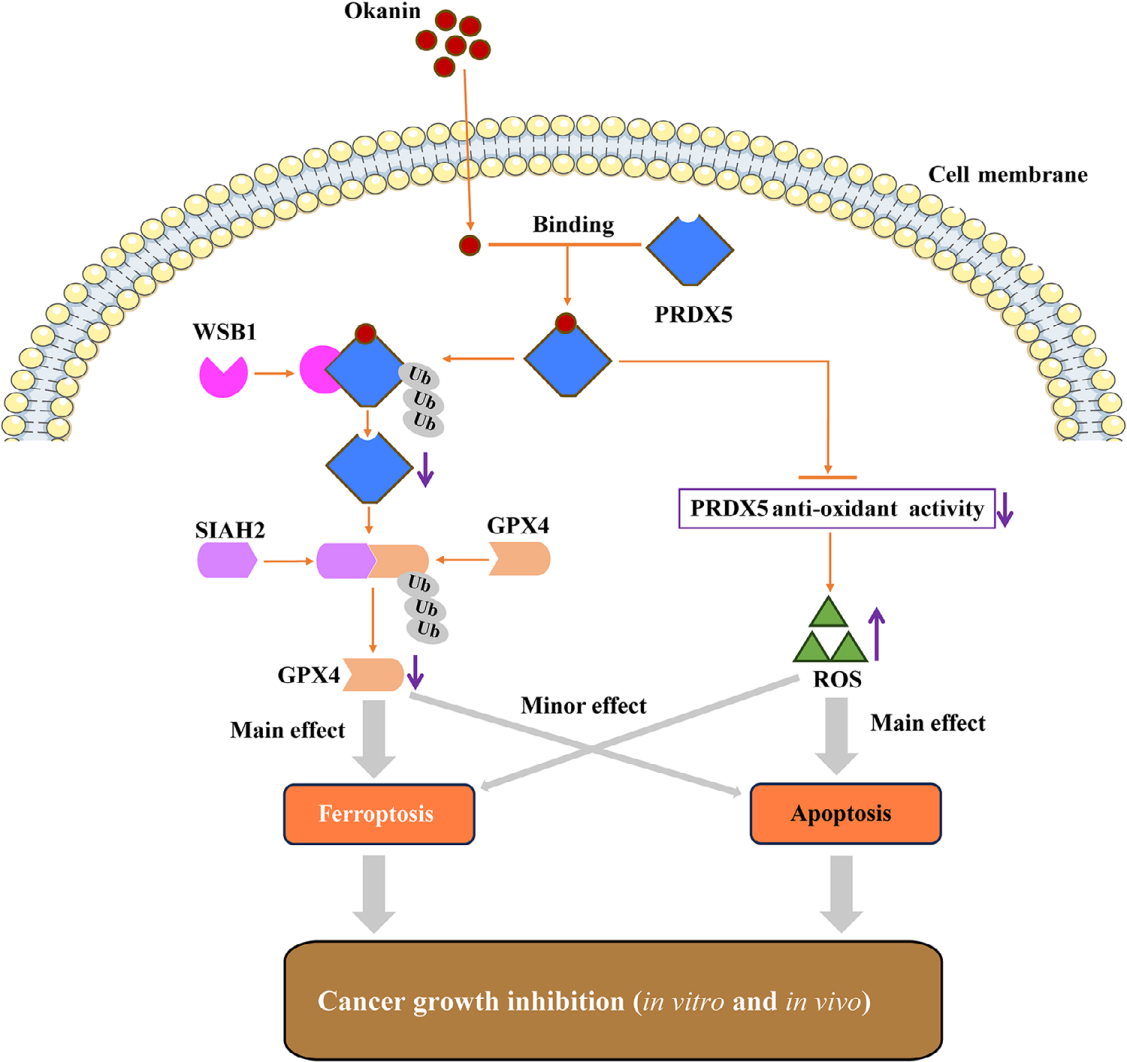
Schematic representation of okanin's anti‐cancer mechanism in colorectal cancer cells. Okanin binds directly to PRDX5, promoting its enhanced interaction with the E3 ubiquitin ligase WSB1, leading to PRDX5 ubiquitination and subsequent proteasomal degradation. The resulting loss of PRDX5 causes reactive oxygen species (ROS) accumulation and SIAH2‐GPX4 axis activation. Ultimately, okanin simultaneously induces ferroptosis and apoptosis, thereby inhibiting cancer cell proliferation/growth.

In conclusion, our results suggest that okanin inhibits the growth of the HCT116 colorectal cancer cells in vitro and in vivo by targeting at and thus suppressing the enzymatic activity of PRDX5 via direct activity inhibition or protein ubiquitination degradation, which results in ROS production and GPX4 ubiquitination degradation that lead predominantly to apoptotic and ferroptotic cell death, respectively (**Figure** **9**). Okanin is a promising candidate drug, and PRDX5 can serve as a drug target for the chemotherapeutic development of treatments of malignancies, including colorectal cancer.

## 5 Experimental Section

### Chemicals, Reagents, Cells and Antibodies

Chemically defined compounds okanin, marein, flavanomarine, ferulic acid, and caffeic acid were purchased from Chengdu DeSiTe Biological Technology Co. Ltd, Chengdu, China. Their purity all reached over 95%, which were dissolved in dimethyl sulfoxide (DMSO) and kept at 4 °C till use. 5‐fluorouracil (5‐Fu), carbonyl cyanide m‐chlorophenylhydrazone (CCCP), Z‐VAD‐FMK, and methyl thiazolyl tetrazolium (MTT) were purchased from Sigma‐Aldrich (St. Louis, MO, USA). The mouse antibodies against PRDX5, ubiquitin, caspase‐3, GPX4, SIAH2, and GAPDH, as well as goat anti‐rabbit and anti‐mouse secondary antibodies, were purchased from Sangon Biotech (Shanghai, China). Colorectal (HCT116), breast (MCF7), and liver (HepG2) cancer cells, as well as immortalized human colon mucosal epithelial (NCM460), bronchial epithelial (BEAS2B), and liver (THLE3) cells, were obtained from the National Collection of Authenticated Cell Cultures (Shanghai, China).

### Cell Culture and Treatment

The cells were cultured in DMEM medium that was supplemented with 10% fetal bovine serum and antibiotics under 37 °C in a humidified incubator with 5% CO_2_. Experiments were performed while the cell density reached ≈70% of confluence. Each of the chemicals was added directly into cell culture to desired working concentration(s) in in vitro experiments. Samples treated for 48 h were used for cell viability assay, while other samples were collected at different time points for Western blot analysis. Colorectal cancer cells treated with okanin for 24 h were used for transcriptomic profiling. In control groups, cells were treated with an equal amount of either medium/solvent DMSO.

### Cell Viability Assay

Cancer cells were seeded into 96‐well plate (6000 cells per well) and cultured for 24 h before treatment for 48 h, after which 10 µL MTT reagent (5 mg mL^−1^) was added to each well and incubated for 4 h at 37 °C in an incubator. Then, the medium was removed and 150 µL DMSO was added to dissolve the crystals (gentle shaking at room temperature for 10–15 min). The absorbance at 490 nm was recorded on a spectrophotometer, which represented the level of cell viability, which reflected the level of cell growth/proliferation.

### Cancer Cell Xenograft Model

Institutional approval was obtained from the Animal Ethics Committee of the Jiangsu Normal University for the experimental protocols. Nude mice (BALB/c nu/nu mice, 6 weeks old) were purchased from the Institute of Laboratory Animals Science (Beijing, China), and were housed in a specific pathogen‐free facility with a 12‐h light/dark cycle, controlled temperature (22 ± 1 °C), humidity (50 ± 5%), and ad libitum access to food and water. After a 7‐day acclimatization, HCT116 cancer cells, as well as their driven cell lines with PRDX5 overexpression (1 × 10^7^ cells mL^−1^, 100 µL) were injected subcutaneously into the left forelimb of each mouse. Mice were randomly assigned intofive groups (n = 5 in each group) for experimetns: 1) PBS control (100 µL day^−1^, i.p.); 2) okanin (20 mg kg day^−1^ in PBS with 5% DMSO, i.p.); 3) 5‐Fu (20 mg kg day^−1^ in PBS, i.p.); 4) PRDX5 overexpression (PBS 100 µL day^−1^, i.p.); and 5) PRDX5 overexpression+ okanin (20 mg kg day^−1^ in PBS with 5% DMSO, i.p.). Treatments commenced 3 days post‐inoculation, with tumor dimensions (length/width) measured every 3 days using a digital caliper (Mitutoyo, Yokohama, Japan) and volume calculated as (length × width^2^)/2. Body weight was also monitored every 3 days via an analytical balance (Sartorius, Göttingen, Germany). After 16 days, the mice were euthanized by CO_2_ inhalation followed by cervical dislocation; the tumors were excised, weighed, and processed for histopathology (4% paraformaldehyde fixation) or molecular analysis (snap‐frozen in liquid nitrogen).

### Potential Target Filtering and Molecular Docking Analysis

PharmMapper server (Version2017; http://www.lilab‐ecust.cn/pharmmapper/) was used to predict okanin's targets. This version applies Cavity1.1 to detect the binding sites on the surface of a given protein structure and rank them according to the corresponding druggability scores. A receptor‐based pharmacophore modeling program Pocket 4.0 was then used to extract pharmacophore features within cavities. When the ligand molecular structure was submitted, a z'score was finally given for its each potential target. The top ten candidates were selected based on the z'score with PRDX5 ranked highest (normalized fit score: 0.98; Table S1, Supporting Information). The molecular docking of okanin to PRDX5 was performed using AutoDock Tools (v1.5.7), with the crystal structure of PRDX5 (PDB ID: 4MMM) retrieved from the Protein Data Bank. The receptor preparation involved removing water molecules and non‐essential ligands using PyMOL (v2.5.2), followed by the addition of polar hydrogens and Kollman charges via AutoDock Tools. The ligand okanin (PubChem CID: 5281294) was prepared by converting its 3D structure from SDF to mol2 format using Open Babel (v3.1.1). Then the mol2 document was converted to PDBQT with AutoDock Tools with hydrogens addition and ligand‐torsion setting. Docking parameters included an exhaustiveness of 20, 20 output modes, and an energy range of 4 kcal mol^−1^. The top‐ranked pose was analyzed for hydrogen bonds and hydrophobic interactions. Docking images were visualized using PyMOL (v2.5.2).

### Quantification of Apoptosis Using Annexin V‐FITC/PI Staining and Flow Cytometry

Sub‐G1 cells were quantified by flow cytometry after staining cells using an annexin V‐FITC/PI apoptosis detection kit (Vazyme Biotechnology, Nanjing, China). The cells were collected at a concentration of 1 × 10^5^ cells mL^−1^, mixed with annexin V‐FITC and PI, and analyzed using a flow cytometer (ACEA Bioscience, San Diego, CA, USA), according to the protocol provided by the manufacturer.

### Quantification of Protein Level Using Western Blot Analysis

Cells were washed twice with cold PBS, lysed with ice‐cold lysis buffer (25 mm Tris·HCl, pH 7.4, 150 mm NaCl, 1 mm EDTA, 1% NP‐40, and 5% glycerol) with protease inhibitors cocktail (Beyotime Biotechnology, Shanghai, China). The lysates were centrifuged at 4 °C for 30 min at 13 000 rpm, and the supernatants were collected. Protein concentrations of the lysates were determined using BCA protein assay kit (Beyotime Biotechnology, Shanghai, China). Cellular proteins were separated by polyacrylamide gel electrophoresis, blotted onto PVDF membrane, which was then blocked with non‐fat milk, washed, incubated with primary antibody, washed, incubated with secondary antibody, and washed. The images of the protein bands were captured using chemiluminescence Imaging System (Clinx, Shanghai, China) and analyzed using Image J software.

### Pull Down Assay for Target Protein of Okanin

The pull‐down assay procedure shown in Figure 2E was performed by covalently conjugating okanin to epoxy‐activated sepharose 6B beads (Biorigin, Beijing, China) through incubation in 0.1 m NaOH coupling buffer (pH 8.0) for 24 h at 25 °C, followed by blocking unreacted sites with 1 m ethanolamine (pH 8.0) for 4 h. Control beads were prepared identically but without okanin conjugation to account for nonspecific interactions. Recombinant human PRDX5 protein (5 µg) or total proteins which extracted from HCT116 cells were incubated with either okanin‐conjugated beads or control beads in binding buffer (20 mm Tris‐HCl, pH 7.4, 150 mm NaCl, 0.1% Triton X‐100) for 6 h at 4 °C with gentle rotation. After five washes with ice‐cold binding buffer to remove unbound proteins, bound PRDX5 was eluted by boiling in 2× Loading buffer and detected via SDS‐PAGE and Western blot using a mouse anti‐PRDX5 antibody (1:1000) and HRP‐conjugated secondary antibody (1:5000), as the description above. Finally, the protein images were captured using chemiluminescence Imaging System and analyzed using Image J software.

### Immunoprecipitation‐Mass Spectrometry (IP‐MS)

Protein complexes were extracted from HCT116 cells co‐expressing His‐tagged PRDX5 and treated with 100 µm okanin for 2 h at 37 °C, followed by lysis in NP‐40 buffer (50 mm Tris‐HCl pH 7.4, 150 mm NaCl, 1% NP‐40, protease inhibitor cocktail) and centrifugation at 13 000 g for 15 min; cleared lysates were subjected to immunoprecipitation overnight at 4 °C using 5 µg anti‐His magnetic beads (Sigma) with gentle rotation, while IgG control samples used equivalent amounts of mouse IgG‐coupled beads; after extensive washing with lysis buffer (3×) and PBS (2×), bound complexes were eluted using 0.1 m glycine‐HCl (pH 2.5) and immediately neutralized with 1 m Tris‐HCl (pH 8.0), followed by tryptic digestion (1:25 enzyme‐to‐protein ratio) in 50 mm ammonium bicarbonate at 37 °C for 16 h; digested peptides were desalted using C18 ZipTips, reconstituted in 0.1% formic acid, and analyzed by nanoLC‐ESI‐MS/MS on a Q‐Exactive HF‐X system (Thermo Scientific, MA, USA) with a 120‐min gradient (2‐35% acetonitrile in 0.1% formic acid) through a 25‐cm PepMap column, operating in positive ion mode with MS1 resolution 70000 (m/z 200–400).

### Transcriptomic Profiling

HCT116 cells were treated with okanin (50 µm) for 36 h, while DMSO and medium were separately used as controls. After the treatment, total RNA was isolated using the Trizol Reagent (Invitrogen Life Technologies, Carlsbad, USA), after which the concentration, quality, and integrity of RNA were determined using a NanoDrop spectrophotometer (Thermo Scientific, CA, USA). Sequencing libraries were then generated and analyzed by Shanghai Personal Biotechnology Cp. Ltd (Shanghai, China). GO enrichment was performed using Gene Ontology database and topGO R language package to analyze the differentially expressed genes; p‐value was calculated by hypergeometric distribution method; and assigned the genes to different terms which were further grouped into categories. KEGG annotation was performed using Cluster Profiler (3.4.4) software for pathways.

### RT‐PCR/qPCR Analyses

RT‐PCR was completed by reverse transcription using M‐MLV reverse transcriptase (Transgene Biotech, Beijing, China). GAPDH was used as a positive and loading control. RT‐PCR products were then examined on 1% agarose gels. The primer sequences used were provided in Table S3 (Supporting Information). qPCR was performed using ViiA 7 Real‐Time PCR System (ThermoFisher, MA, USA) with TransStart Top Green qPCR SuperMix (Transgene Biotech, Beijing, China). mRNA expression was normalized to the reference housekeeping gene, GAPDH.

### Overexpression of Protein Using Plasmid

The entire coding region of the concerned genes was obtained via reverse transcription PCR from the total RNA, which was subcloned into the pCDNA3.1 plasmid vector using the *KpnΙ/BamHI* restriction enzymes. The primer used in the reverse transcription PCR were presented in Table S4 (Supporting Information). The construct was transformed into *E. coli* DH5α for amplification, which was then sequenced by Beijing Genomics institution (BGI, Beijing, China) to confirm the correct cloning. The new constructed plasmids were transfected into HCT116 cells using Lipo2000 (Thermo Fisher Scientific, MA, USA). Lipo2000 or the plasmids alone was pre‐incubated in DMEM without FBS for 5 min, followed by mixing the two and incubation for 20 min, which was added to the cell culture for the transfection for 36 h. At the end, the cells were lysed, and the total mRNA and proteins were extracted for quantification of the level of mRNA and proteins of interest using qPCR and Western blot analysis.

### Knockdown and Knockout Cells Construction

siRNA species of PRDX5, WSB1, GPX4, and SIAH2 were purchased from GenePharam company (Shanghai, China). The sequences were listed in Table S5 (Supporting Information). For siRNA transfection, HCT116 cells (5 × 10^4^ cells per well) were seeded in six‐well plates and cultured for 24 h, and then transfected with 50 nm siRNA oligonucleotides using siRNA‐Mate transfection reagent (GenePharam), according to the manufacturer's instructions. The protein level was evaluated by Western blot analysis after 24 h of transfection.

To generate stable WSB1 and SIAH2 knockout HCT116 cell lines, the pX459‐CRISPR‐Cas9 plasmid was employed as the backbone for sgRNA delivery. Two target‐specific sgRNAs per gene (Table S6, Supporting Information) were designed using the CHOPCHOP algorithm and synthesized as oligonucleotides by Sangon Biotech (Shanghai, China). These sgRNA oligos were annealed and cloned into the BbsI restriction site of the pX459 vector, which co‐expresses Cas9 and a puromycin resistance gene. HCT116 cells were transfected with the constructed sgRNA‐Cas9 plasmids using Lipo2000 (Thermo Fisher Scientific, MA, USA) according to the manufacturer's protocol. At 48 h post‐transfection, cells underwent puromycin selection (2 µg mL^−1^) for 72 h to eliminate non‐transfected cells. Surviving cells were subjected to monoclonal expansion, followed by Western blotting assays to confirm complete protein ablation.

### Quantification of ROS Using Flow Cytometry

Intracellular ROS was quantified in a FACS Caliber flow cytometer (Becton Dickinson) using the peroxide‐sensitive fluorescent probe dichlorofluorescein diacetate (DCFH‐DA; Beyotime, Shanghai, China) according to the manufacturer's instructions. Briefly, HCT116 cells were incubated for 20 min at 37 °C with 10 µm DCFH‐DA diluted in serum‐free medium, washed three times with serum‐free medium, resuspended in ice‐cold PBS, and kept in the dark shortly before flow cytometry analysis. ROS level was quantified at an emission wavelength of 525 nm. At least 10000 individual cells were acquired for each group using CellQuest Software (BD Biosciences) and analyzed using FlowJo 7.6 software (TreeStar).

### Assay of Trx‐TrxR‐NADPH Coupling Peroxidase Activity In Vitro

The assay was conducted in 96‐well plates as previously described.^[^ ^20^ ^]^ Each of the natural compounds was incubated with PRDX5 for 1.5 h at room temperature. A solution containing 0.3 µm yeast Trx, 0.15 µm yeast TrxR, and 200 µm NADPH was added to each well to initiate the reaction. An aliquot of 100 µm hydrogen peroxide (H_2_O_2_) was added, followed by detection of optical density by Thermo Multiskan FC at 340 nm; the decrease in the optical density represented the enzymatic activity level. Concentrations of PRDX5 and each of the compounds were set at 0.6, 1.5, 1, 3, 5, and 10 µm.

### Quantitation of Lipid Hydroperoxide

LPO was quantitated using LPO Content Assay Kit (Solarbio Life Science, Beijing, China). Briefly, LPO Samples were first extracted from the sample into chloroform. Then, 500 µL of chloroform extract, 450 µL of chloroform‐methanol solvent, and 50 µL of chromogen solution (equal volumes of FTS reagent 1 and FTS reagent 2) were mixed with each sample. The reactions were incubated at room temperature for 5 min and read on a spectrophotometer at 500 nm (Microplate reader, Epoch, BioTek, USA). Optical density represented the level of LPO.

### Quantitation of GSS and GSSG

GSH and GSSG contents were detected using GSH and GSSG Assay Kit (Beyotime Biotechnology, Nantong, China), respectively, according to the manufacturer's protocols. Cells were twice frozen with liquid nitrogen and thawed in a water bath at 37 °C, and centrifuged at 10 000 g for 10 min at 4 °C. Supernatants were used for determination of GSH and GSSG. Absorbance was measured at 450 nm using a microplate reader (BIO‐DL, Shanghai, China). Optical density represented their levels.

### Quantitation of Free Fe^2+^

Intracellular Fe^2+^ level was analyzed by FerroOrange staining kit (MaoKang Biological Science, Shanghai, China). Cells were seeded at 1 × 10^5^ cells per well into 12‐well plates, cultured, and then treated. FerroOrange probe (1 µm) was added for 30 min. Then cells were washed twice with PBS. Fluorescence image was captured using Leica biological microscope DM6000B (Leica, Weztlar, Germany); green laser (ex. 532 nm) was used to excite FerroOrange.

### Microscale Thermophoresis Assay

Microscale thermophoresis (MST) assay was performed to determine the binding between okanin and PRDX5. In brief, purified protein of PRDX5 was labeled with fluorescence using a GREEN‐NHS protein labeling kit (NanoTemper, Germany) according to manufacturer's protocol. Each experiment used 16 samples: the concentration of fluorescently labeled PRDX5 protein remained constant, and okanin was used at concentrations with a 2‐fold increment. Okanin and PRDX5 were mixed and incubated for 30 min at room temperature. Then, mixtures were loaded and measured using a Monolith NT.115 instrument (Nano Temper Technologies GmbH, Munich, Germany) at 25 °C with 20% excitation power and 40% MST power. The temperature‐induced changes in fluorescence (temperature‐related intensity changes and/or temperature‐dependent movements) were determined as a function of PRDX5 target probe concentration in glass capillaries. Fnorm = F1/F0 (Fnorm: normalized fluorescence; F1: fluorescence after thermodiffusion; F0: initial fluorescence or fluorescence after T‐jump). Dissociation constant (*Kd*) was calculated and fitted by Nano Temper Analysis software. Three independent measurements were analyzed using the signal from thermophoresis plus T‐Jump.

### Statistical Analysis

The experiments were performed in triplicate. All data were represented as mean ± SD. Statistical analysis was performed using SPSS computer software package. Student's t‐test was used for the comparison between two groups. Multi‐group comparisons were performed using one way ANOVA. A *p* <0.05 was considered to be statistically significant. ^*^: *p* < 0.05; ^**^: *p <* 0.01; ^***^: *p* < 0.001, compared with the control group. ^#^: *P* < 0.05; ^##^: *P* < 0.01; ^###^: *P* < 0.001, compared with the treated group. All experiments were repeated three times independently.

### Ethical Approval Declaration

Adherence to ARRIVE guidelines for animal studies was confirmed, and the animal experimental approval was obtained from the Animal Ethics Committee of Jiangsu Normal University (JSNU‐IACUC‐2024039).

## Conflict of Interest

The authors declare no conflict of interest.

## Supporting information



Supporting Information

Supporting Information

## Data Availability

The data that support the findings of this study are available from the corresponding author upon reasonable request.
