# Supplementary material for: Okanin Suppresses the Growth of Colorectal Cancer Cells by Targeting at Peroxiredoxin 5
Source: Adv Sci (Weinh). 2025 Aug 20;12(43):e17148. doi: 10.1002/advs.202417148 (PMC12631902; doi:10.1002/advs.202417148)
Supplement: Supplementary file 1 — Supporting Information [file ADVS-12-e17148-s001.pdf]

Supplementary information

Table 1. The candidate target proteins of okanin.

Protein name	Score
Peroxiredoxin-5	9.0
Complement factor B	8.1
Stromelysin-1	6.3
Mitogen-activated protein kinase 10	7.0
Glutathione S-transferase P	5.2
Mitogen-activated protein kinase 8	5.4
Tyrosine-protein phosphatase non-receptor type 1	6.1
Prothrombin	5.1
Neutrophil gelatinase-associated lipocalin	4.3
Liver carboxylesterase 1	6.2

Table 2. The transcriptomic profiling data (provided as a separate document)

Table 3. List of primers used for quantification of gene expression

Gene	*Forward (5'-3')*	*Reverse (5'-3')*
**PRDX5**	TGATTCGCTGGTGTCCATCTTTG	GTGCCATCTGGTTCCACATTCAGG
**WSB1**	TGTTCCGTGGTCCCAGTGCCTTCA	CGCCCATTGTTCAACCCTGTAGC
**GPX4**	TCGGGAAGCAGGAGCCAGGGAGTA	GCAGCCGTTCTTGTCGATGAGGA
**SIAH2**	CGGCAGTCCTGTTTCCCTGTAAGT	GAAGGGTGGTAATGCTCTTGTGGG

Table 4. List of primers used for overexpression vector construction.

Plasmid name	*Gene*	*Forward (5'-3')*<br>*Reverse (5'-3')*	*Restriction enzyme*
**pCDNA3.1-his**	PRDX5	CGGAATTCATGGGACTAGCTGGCGTGTGCGCCCT<br>TTAAGGTACCGAGCTGTGAGATGATATTGGGTG	*Eco*R I<br>*Kpn* I
**pCDNA3.1-his**	WSB1	CGGAATTCATGGCCAGCTTTCCCCCGAGGGTCA<br>TTAAGGTACCAATACGATACGAGAGAAACTCCA	*Eco*R I<br>*Kpn* I
**pCDNA3.1-his**	GPX4	CGGAATTCATGAGCCTCGGCCGCCTTTGCCGC<br>TTAAGGTACCTGAGTGCCGGTGGAAGGCTCCAAG	*Eco*R I<br>*Kpn* I
**pCDNA3.1-his**	SIAH2	CGGAATTCATGAGCCGCCCGTCCTCCACCGGC<br>TTAAGGTACCTGGACAACATGTAGAAATAGTAACA	*Eco*R I<br>*Kpn* I
**pCDNA3.1-his**	SMURF2	CGGAATTCATGTCTAACCCCGGAGGCCGGAGGA<br>TTAAGGTACCTTCCACAGCAAATCCACATGTTTCT	*Eco*R I<br>*Kpn* I

Table 5. List of siRNA sequences used in this study.

siRNA	*Oligomers (5'-3')*
**PRDX5**	GGAAUCGACGUCUCAAGAGGUTT
**WSB1**	GAAAACUCCUCCUUAACUUTT

**GPX4**	GAGGCAAGACCGAAGUAAATT
**SIAH2**	CAUCGUCUUUCUAGCUACATT
**USP1**	GCAUAGAGAUGGACAGUAUTT
**UBA6**	CCTTGGAAGAGAAGCCTGATGTAAA

Table 6. List of sgRNA sequences used in this study.

sgRNA	*Target Sequence (5'-3')*
**WSB1**	TATGATCTACCAAGTTAAGGAGG
**SIAH2**	ACGCCCCGTTCGATTCATGACGG

Supplementary Figures

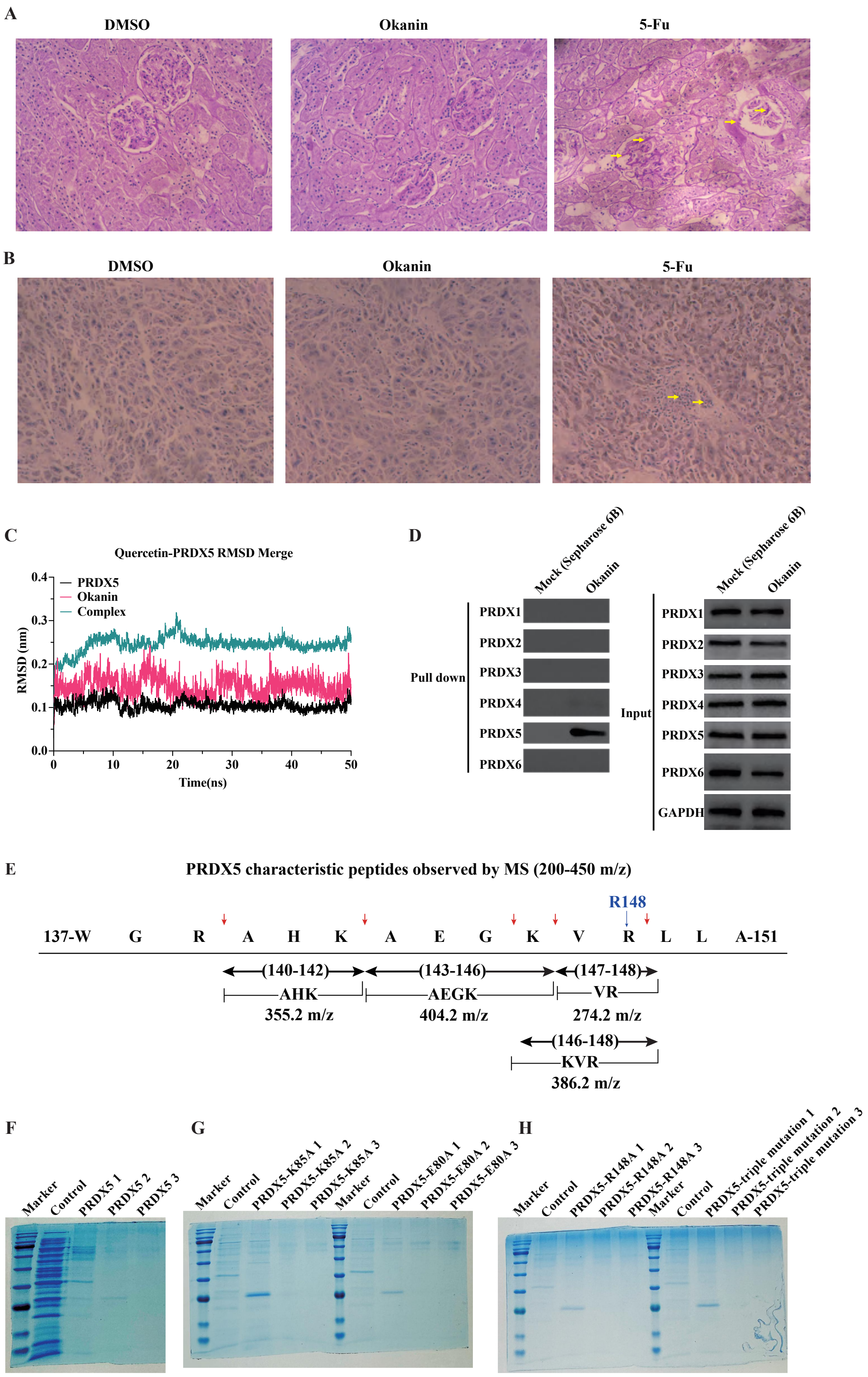

Figure 1. (A) 5-Fu-treated mice exhibited mesangial matrix deposition and mesangial expansion, whereas these pathological features were absent in okanin-treated counterparts (yellow arrows). (B) Hepatic analysis indicated inflammatory infiltrates in 5-Fu-administered mice (yellow arrows), contrasting with minimal or no inflammatory reactions observed in both control and

okanin intervention groups. (C) Molecular dynamics simulations analysis of okanin-PRDX5 complex. Root Mean Square Deviation (RMSD) value < 0.5 over 50 ns. (D) Epoxy-activated Sepharose 6B-immobilized okanin confirmed specific binding to PRDX5, while other PRDX isoforms showed minimal interaction. (E) This diagram illustrates tryptic cleavage sites (red arrows) and critical residues (blue highlight at R148) in PRDX5's sequence (residues 137-W to 151-A), featuring observed peptides: AHK (140–142), 355.2 m/z; AEGK (141–144), 404.2 m/z; KVR (146–148), 386.2 m/z; VR (147–148), 274.2 m/z. The KVR peptide forms due to failed tryptic cleavage at R148, whereas VR peptide requires cleavage at R148; okanin's occupation of the R148 site via hydrogen bonding may blocks trypsin access, suppressing VR generation while promoting accumulation of the uncleaved KVR peptide, conclusively proving okanin’s specific engagement with PRDX5. (F) The purification of wild type PRDX5. (G and H) The purification of PRDX5 mutation at K85A, E80A, R148A and triple mutation.

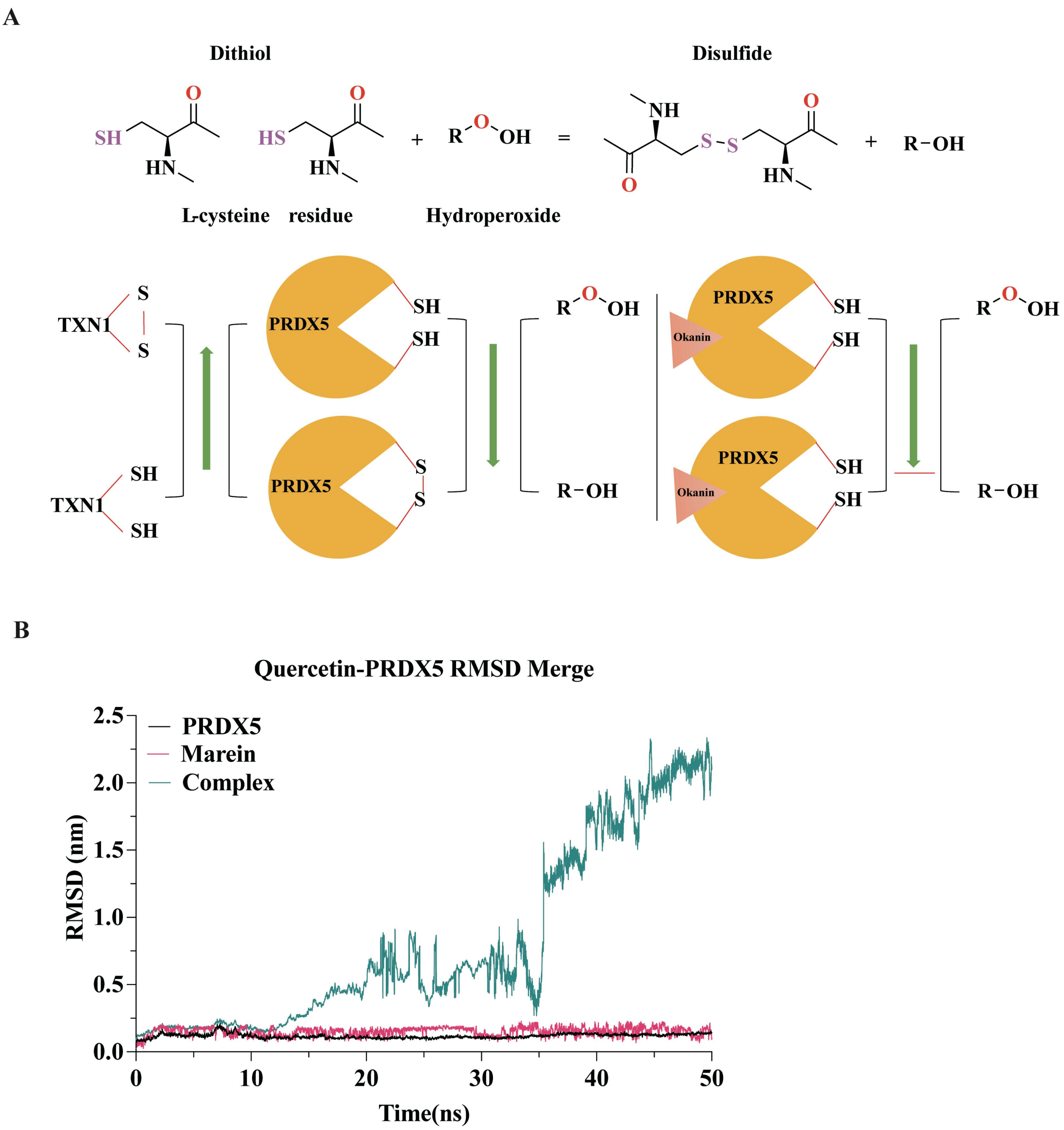

Figure 2. (A) Diagram of ROS reduction by PRDX5. (B) Molecular dynamics simulations analysis of marein-PRDX5 complex. Root Mean Square Deviation (RMSD) value >0.5 at 20 ns and >2.0 at 40 ns.

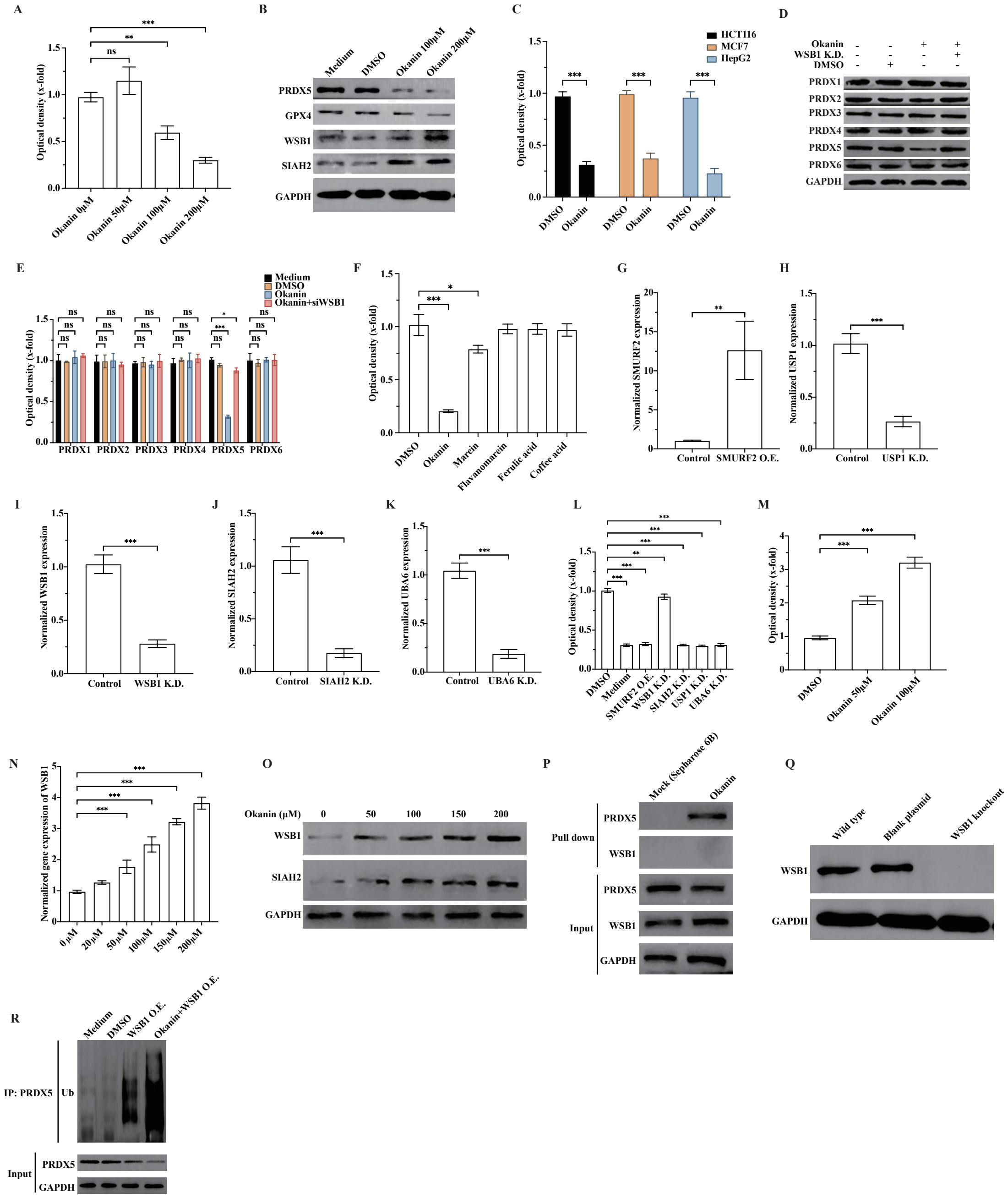

Figure 3. (A) Quantitative densitometric analysis of western blot results for the indicated panels in Fig. 4A. (B) Okanin dose-dependently downregulated PRDX5 protein expression while upregulating WSB1 and SIAH2 levels in SW480 cells. (C) Quantitative densitometric analysis of Western blot results for the indicated panels in Fig. 4B. (D and E) Western blot analysis of PRDX family protein expression profiles following okanin treatment, WSB1 knockdown, or their combination. (E and F) Quantitative densitometric analysis of Western blot results for the indicated panels in Fig. S3D and Fig. 4C, respectively. (G) qPCR analysis of SMURF2 overexpression following plasmid transfection. (H-K) qPCR analysis of siRNA-mediated knockdown of USP1 (H), WSB1 (I), SIAH2 (J), and UBA6 (K). (L and M) Quantitative densitometric analysis of western blot results indicated panels in Fig. 4G and Fig. 4I,

respectively. (N) Okanin dose-dependently increased WSB1 expression in mRNA level. (O) Okanin dose-dependently increased WSB1 and SIAH2 expression in protein levels. (P) Okanin-coupled Sepharose 6B beads can pull down PRDX5 but not WSB1. (Q) WSB1 was stably knocked out in HCT116 cells. (R) WSB1 overexpression enhanced PRDX5 ubiquitination. ns: no significant; *: $p<0.05$; **: $p < 0.01$; ***: $p < 0.001$.

A

##		WSB1				×	PRDX5				interface	$\Delta^{i}G$	$\Delta^{i}G$	N_{HB}	N_{SB}	N_{DS}	CSS
NN	«»	Range	$^{i}N_{at}$	$^{i}N_{res}$	Surface Å^2		Range	$^{i}N_{at}$	$^{i}N_{res}$	Surface Å^2	area, Å^2	kcal/mol	P-value				
1	◉	A	115	37	21063	◊	B	106	29	7372	1046.3	−15.4	0.048	9	1	0	0.420

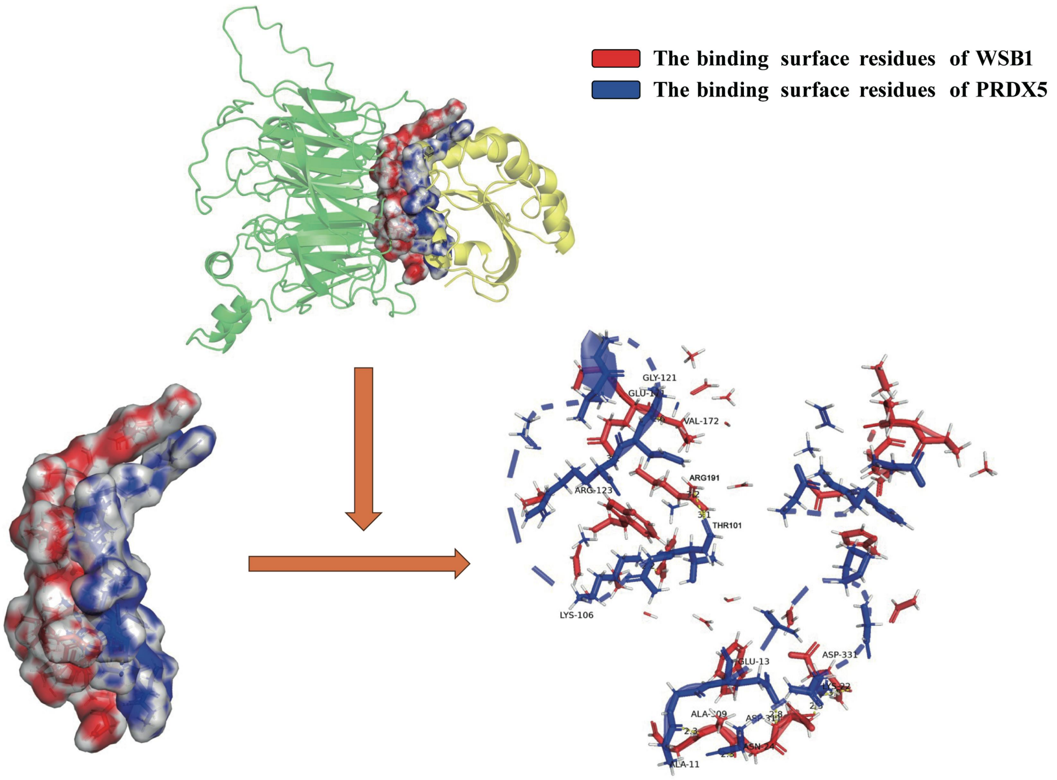

B

##		SIAH2				×	GPX4				interface	$\Delta^{i}G$	$\Delta^{i}G$	N_{HB}	N_{SB}	N_{DS}	CSS
NN	«»	Range	$^{i}N_{at}$	$^{i}N_{res}$	Surface Å^2		Range	$^{i}N_{at}$	$^{i}N_{res}$	Surface Å^2	area, Å^2	kcal/mol	P-value				
1	◉	A	133	46	18493	◊	C	113	31	8449	1240.3	−19.3	0.020	14	0	0	0.460

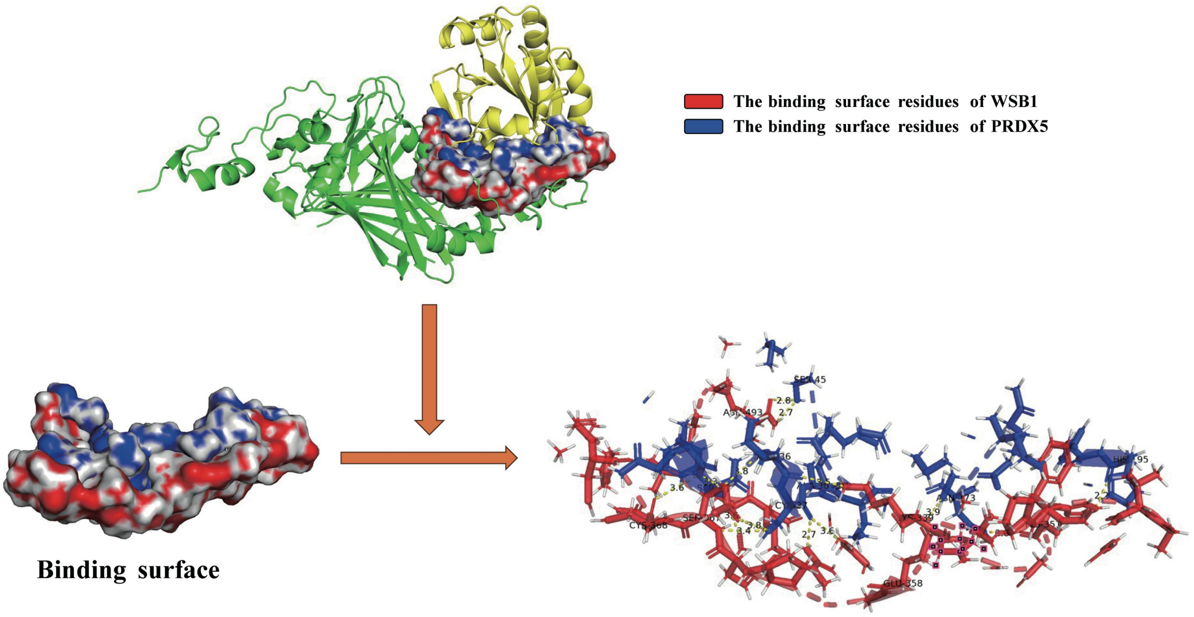

Figure 4. (A) The molecular docking diagram of WSB1 and PRDX5. The binding interface between WSB1 and PRDX5 involves 37 residues on WSB1 and 29 residues on PRDX5, stabilized by nine hydrogen bonds involving key residues ALA309 [N], ARG191 [NH2],

ARG191 [NH1], TYR218 [OH], VAL172 [N], ASP331 [O], ASP311 [O], ALA 309 [O] and TYR 218 [OH] from WSB1 with ALA11 [O], PRO100 [O], THR101 [O] , GLY121 [O] , LYS22 [NZ], LYS22 [NZ], ASN24 [ND2], and LYS 106 [NZ] from PRDX5, respectively (Fig. S7A). The interface area of WSB1 and PRDX5 is 1046.3 $Å^2$, and the Δ^iG is -15.4 kcal/mol. (B) The molecular docking diagram of SIAH2 and GPX4. The binding interface between SIAH2 and GPX4 involves 46 residues on SIAH2 and 31 residues on GPX4, stabilized by fourteen hydrogen bonds involving key residues LYS 339 [NZ], ARG 364 [NH2], TYR 340 [OH], SER 367 [N], LYS 339 [NZ], ASN 493 [ND2], HIS 351 [NE2], CYS 368 [O], ILE 483 [O], SER 367 [OG], GLN 378 [OE1], ASN 493 [OD1], HIS 351 [O], and GLU 358 [OE1] from SIAH2 with ARG 36 [O], CYS 37 [O], CYS 37 [O], CYS 37 [SG], ALA 38 [O], SER 45 [OG], HIS 195 [O], SER 34 [N], SER 34 [OG], ARG 36 [N], CYS 37 [SG], SER 45 [OG], and ASN 173 [ND2] from GPX4, respectively. The interface area of SIAH2 and GPX4 is 1240.3 $Å^2$, and the Δ^iG is -19.3 kcal/mol.

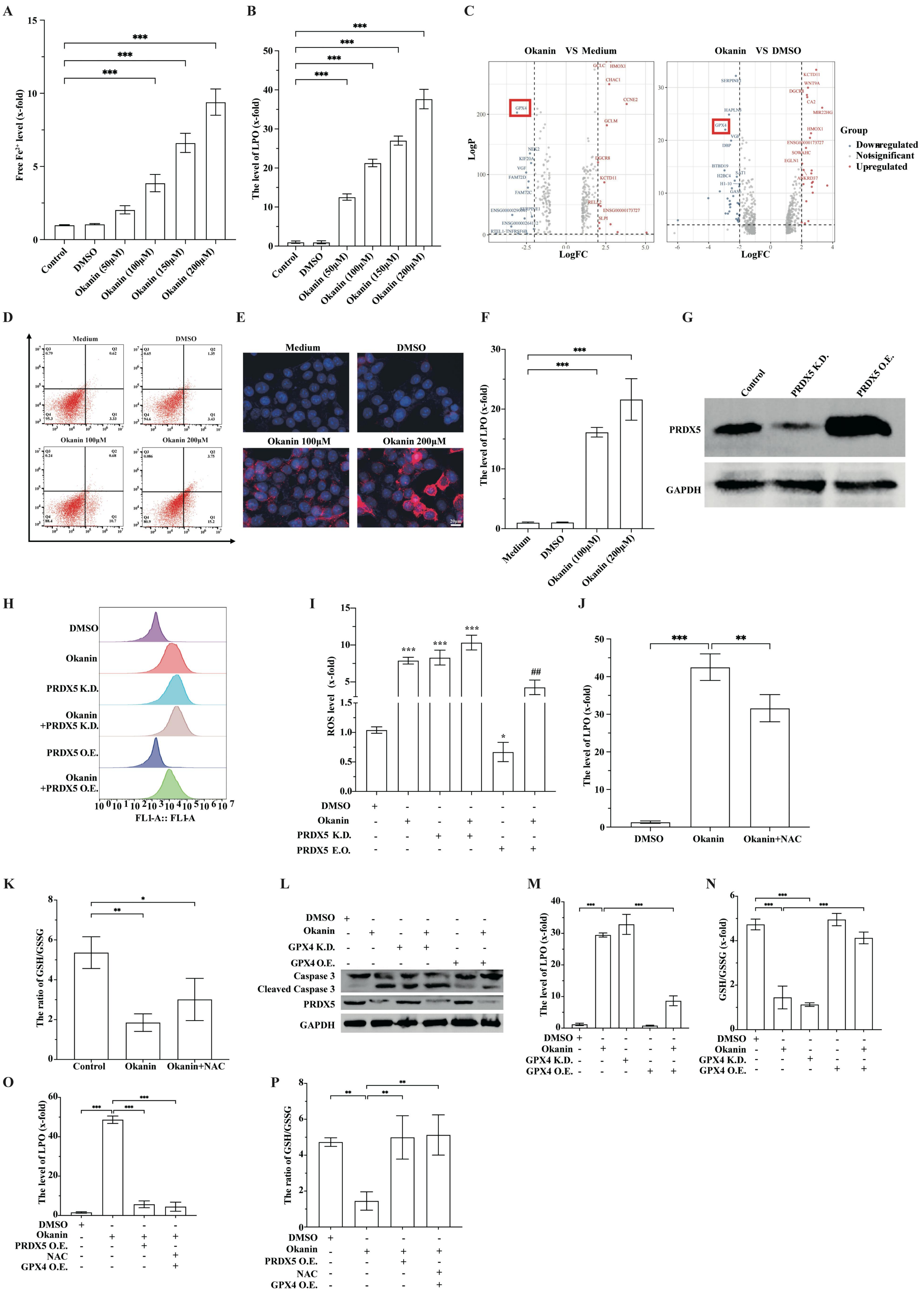

Figure 5. (A and B) Okanin upregulated Free Fe^{2+} and LPO levels in a concentration dependent manner following 36 h treatment. (C) The transcriptomic profiling results revealed that okanin inhibited GPX4 transcription compared to both medium and DMSO group. (D) Okanin induce

apoptosis in dose-dependent manner as validated by Annexin V/PI dual staining. (E and F) Okanin induced ferroptosis in HCT116 in a dose-dependent manner as evidenced by elevated intracellular ferrous iron (FerroOrange fluorescence, E) and LPO (Liperfluo fluorescence, F) levels. (G) PRDX5 overexpression and knockdown analysis using western blot. (H) PRDX5 knockdown promoted ROS production, similarly to okanin-treatment, whereas PRDX5 overexpression inhibited okanin-increased ROS production. (I) The quantitation of ROS in Fig. S5H. (J) NAC inhibited okanin-increased LPO. (K) NAC also reversed the okanin-induced decrease in the GSH/GSSG ratio, although not statistically significantly. (L) GPX4 overexpression or knockdown did not affect okanin induced caspase 3 activation. (M and N) GPX4 overexpression largely inhibited okanin-induced upregulation of LPO (M) and also reversed the okanin-mediated decrease in the GSH/GSSG ratio (N). (O and P) The combination of NAC and GPX4 overexpression resulted in a nearly complete blockage of okanin-promoted LPO (O) and a decrease in the ratio of GSH/GSSG (P), both of which were similar to the effects of PRDX5 overexpression alone. ns: no significant; *: p<0.05; **: p < 0.01; ***: p < 0.001.

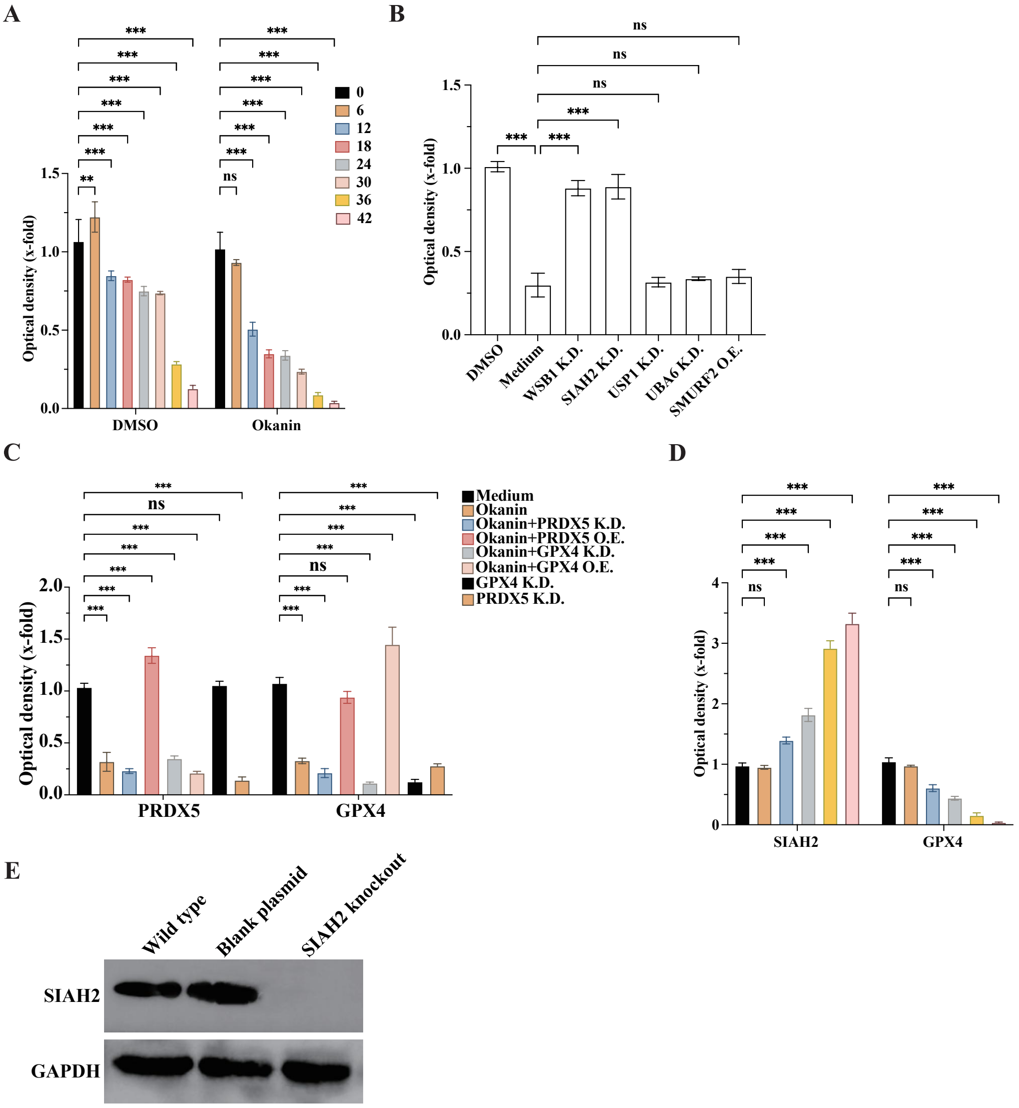

Figure 6. (A-D) Correspond to quantitative densitometric analysis of Western blot results for the indicated panels in Figs 7A, 7C, 7D, and 7E. (E) SIAH2 was stably knocked out in HCT116 cells. *: p<0.05; **: p < 0.01; ***: p < 0.001.

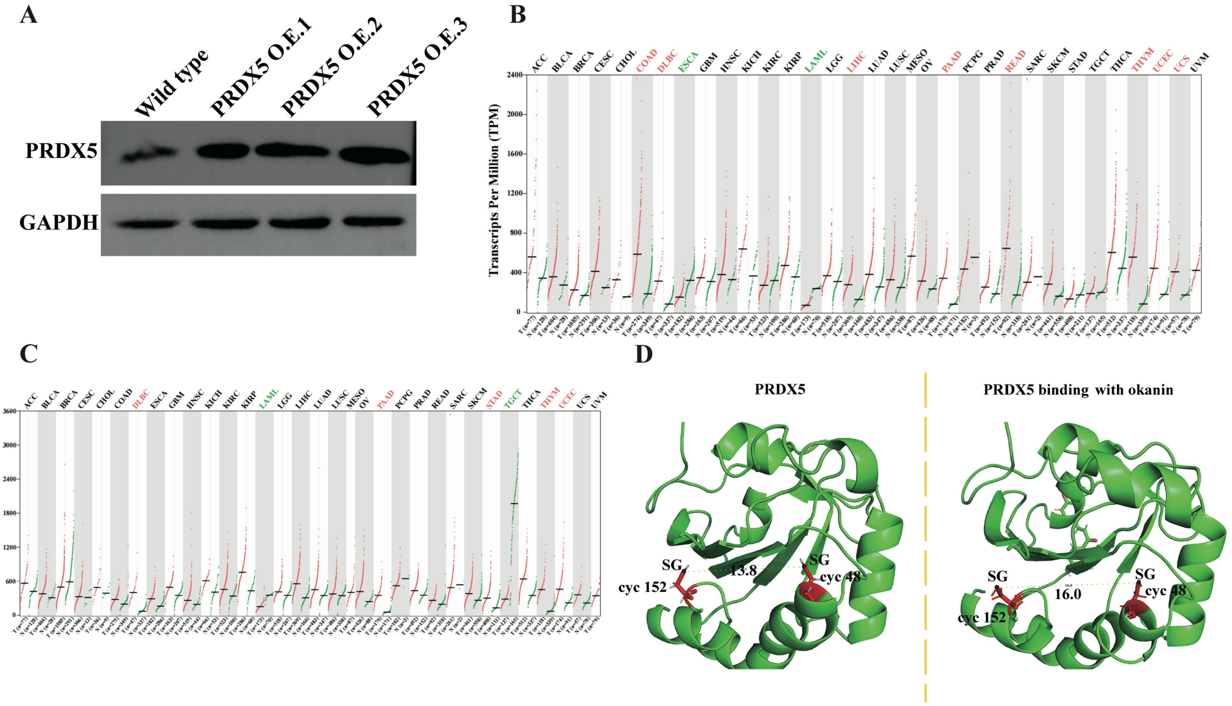

Figure 7. (A) PRDX5 stably expressed in HCT116 cells. (B and C) The expression profiling of PRDX5 (B) and GPX4 (C) in different cancer patients compared to healthy subjects. Red: increased; Green: decreased; and Black: no difference. (D) Okanin binding induces a conformational shift in PRDX5's thioredoxin fold. This shift increases the distance between the catalytic Cys48-SH and Cys152-SH residues from 13.8 Å (apo-PRDX5) to 16 Å (okanin-bound), accompanied by a near-180° rotation of the sulfur-bound hydrogen atoms.
